# Two-stage multiple imputation with a longitudinal composite variable

**DOI:** 10.1186/s12874-025-02555-9

**Published:** 2025-05-06

**Authors:** Xuzhi Wang, Martin G. Larson, Chunyu Liu

**Affiliations:** Department of Biostatistics, Boston University School of Public Health, Boston, MA 02118 USA

**Keywords:** Multiple imputation, Missing data, Missing not at random, Composite variable

## Abstract

**Background:**

Missing data are common in longitudinal studies. Multiple imputation (MI) is widely used to handle missing data. However, most of the MI methods assume various missing data types as missing at random (MAR) in imputation. Two-stage MI is a flexible method that accounts for two types of missing data in a two-step process, allowing researchers to employ diverse assumptions regarding the mechanisms underlying the missing data. This method has immense potential yet limited application and extension within the field.

**Methods:**

We evaluated the performance of two-stage MI in a novel context, imputing a composite variable constructed from several continuous and binary components in the longitudinal setting while handling missing data due to MAR and missing not at random (MNAR). Additionally, we compared three fully conditional specification (FCS) methods within the two-stage MI framework. Simulation studies were conducted using a longitudinal dataset that mimicked a cohort study. Sensitivity analysis was performed with various ignorability assumptions.

**Results:**

In simulation studies, the imputation models within two-stage MI, assuming appropriate ignorability assumptions, exhibited the smallest bias and achieved optimal coverage probabilities for the means, slopes across different time points, and hazard ratios for mortality related to the composite variable. The FCS methods that incorporated longitudinal information yielded the best performance in most scenarios.

**Conclusions:**

In the context of a longitudinal composite variable with missing values due to various missing mechanisms, the selection of imputation methods and ignorability assumptions plays an important role within the two-stage MI framework.

**Supplementary Information:**

The online version contains supplementary material available at 10.1186/s12874-025-02555-9.

## Introduction

Missing data are frequently encountered in biomedical research, especially in longitudinal studies [1]. In such studies, missingness can have different patterns, such as dropout (i.e., monotone missingness) and intermittent missingness [2]. Furthermore, the missingness in incomplete data may result from different underlying mechanisms, i.e., missing completely at random (MCAR), missing at random (MAR), and missing not at random (MNAR). Missing data mechanisms can also be categorized as ignorable and non-ignorable [1].

Multiple imputation (MI) provides a flexible framework for handling missing values [2–4]. MI creates multiple datasets to fill in plausible values for each of the missing data points. Each imputed dataset is individually analyzed to compute estimates of interest. These estimates are later combined using appropriate combining rules. Missing data may result from more than one missing mechanisms in a longitudinal dataset [5]. However, conventional MI methods usually assume missing data as MAR, treating various types of missing data using a uniform approach regarding their underlying mechanisms.

Two-stage MI (or nested MI) is a method that accounts for two types of missingness in a two-step process [3, 6, 7]. The general idea of two-stage MI is to impute the first type of missingness $m$ times in the first stage. For each of the $m$ datasets generated in the first stage, imputation fills in the second type of missing values $n$ times, treating the imputed values of the first type of missingness as fixed and known. One advantage of two-stage MI is the flexibility to apply different ignorability assumptions to each type of missingness. This method has been applied to scenarios where missing values due to both MAR and MNAR are assumed [7], and it has also been utilized to account for the uncertainty of the missing data mechanisms by incorporating a range of ignorability assumptions using the two-stage combining rules [8, 9].

Previous two-stage MI research mainly focuses on one continuous or binary variable with missing data due to MAR or MNAR. One area yet to be studied involves evaluating two-stage MI in the context of a longitudinal composite variable that is generated by combining two or more individual components [10]. To that end, we aim to extend and evaluate the two-stage MI framework in a novel context. We use a longitudinal composite variable for imputation, addressing both MAR and MNAR missing mechanisms. The composite variable comprises both continuous and binary individual components. Additionally, we aim to apply and compare several fully conditional specification (FCS) methods within the two-stage MI framework. Prior studies investigate the two-stage MI framework based on only one imputation method [3, 7, 8, 11]. In real data application, we apply our extension of the two-stage MI framework in a survival-analysis setting and compare it to other survival models using the available data.

## Methods

### The Framingham Heart Study—a motivating example

The Framingham Heart Study (FHS) is a multigenerational cohort study that began in 1948 to identify risk factors for cardiovascular disease (CVD), with the Original cohort enrolling 5209 men and women from Framingham, Massachusetts [12]. In 1971, the Offspring cohort enrolled 5214 participants who were the offspring of the Original cohort and the spouses of these offspring [13]. The participants in the Offspring cohort have been examined every 4 to 8 years. One previous study investigated the FHS Offspring cohort from exam 5 to exam 8 by applying MI methods on healthy aging index (HAI), a composite score that predicts subclinical disease in older populations [14]. Simulation studies have shown that MI methods produced biased results for HAI when missing data were MNAR.

To evaluate cardiovascular health in the FHS Offspring cohort across exams, we propose a new longitudinal composite variable, referred to as Composite-5, incorporating five continuous and binary components. The five components include smoking status, body mass index (BMI), blood pressure (BP) [both systolic BP (SBP) and diastolic BP (DBP)], total cholesterol, and fasting blood glucose. Except for smoking status (1 for smokers and 0, otherwise), the other four variables are continuous. Detailed steps for constructing Composite- 5 are summarized in Supplementary Material 1: Table S1.

Each of the five component is dichotomized to create a binary indicator before constructing Composite- 5. Of note, higher levels of individual components reflect suboptimal health or disease, which correspond to lower binary individual indicators that lead to lower Composite- 5 for worse cardiovascular health. In contrast, higher Composite- 5 values indicate better cardiovascular health. Composite- 5 is motivated by Life’s Simple 7, which further includes physical activity and diet [15]. These two components were excluded from our new composite variable because they were collected at fewer exams through ancillary study funding.

### Data setup and analysis models of interest

Let ${C}_{it}$ be the value of a longitudinal composite variable for subject $i$ $(i=\text{1,2},\dots ,N)$ at time $t$ $(t=\text{1,2},\dots ,T)$. $N$ denotes the total number of participants, and $T$ denotes the number of repeated measurements of the longitudinal composite variable. Suppose ${C}_{it}$ is constructed based on multiple binary and continuous components ${Y}_{it}^{p}$ $(p=\text{1,2},\dots ,P)$. $P$ represents the number of components for constructing the composite variable. Let ${Y}_{i}^{p}=({Y}_{i1}^{p}, {Y}_{i2}^{p}, \dots , {Y}_{iT}^{p})$,${Y}_{i}=({Y}_{i}^{1}, {Y}_{i}^{2}, \dots , {Y}_{i}^{P})$, and $Y=({Y}_{1}, {Y}_{2}, \dots , {Y}_{N})$. $Y$ represents the vector of $P$ individual components for all participants. Note that ${C}_{it}={\Sigma }_{p=1}^{P}{D}_{it}^{p}$, where the binary indicator $${D}_{it}^{p}=\left\{\begin{array}{c}1, \text{if} \; {Y}_{it}^{p} \; \text{is} \; \text{ideal}\\ 0, \text{if} \; {Y}_{it}^{p} \; \text{is} \; \text{not} \; \text{ideal}\end{array}\right.$$. The criteria for “ideal” and “not ideal” in the binary indicator depend on the specific composite variable being studied.

We focus on three key questions: (i) how well does two-stage MI preserve the average values of the composite variable at each time point? (ii) how accurately does two-stage MI capture the temporal trend of the composite variable? (iii) how well does two-stage MI recover the predictive ability of the composite variable for a time-to-event outcome (e.g., mortality) in the survival analysis? To answer the three questions, we evaluate the imputed composite variable using three estimators: the mean of the composite variable at each time point for question (i), the slope of the composite variable across time points in the linear mixed model for question (ii), and the hazard ratio (HR) of the time-to-event outcome for each unit increase in the composite variable in the Cox model for question (iii). The primary goal of the simulation study is to identify the imputation method and ignorability assumption that optimizes the performance of these three estimators within the two-stage MI framework. We evaluate the imputed composite variable using three statistical estimators: the mean of the composite variable at each time point, $\overline{{C }_{t}}$; the slope of the composite variable estimated from the linear mixed model, ${\beta }_{1}$; and the log hazard ratio for each unit increase in the composite variable estimated from the Cox model, ${\gamma }_{1}$. These estimators are specified as follows:

- Mean: For each imputed dataset, we calculate the means of the composite variable across all participants at each time point $t$: $\overline{{C }_{t}}={\sum }_{i=1}^{N}\frac{{C}_{it}}{N}$.
- Slope: To capture the trend of the composite variable across time points, we conduct linear mixed models with the composite variable as the repeated outcome and time as the main predictor. Let ${{\varvec{X}}}_{{\varvec{i}}}$ be a vector of baseline covariates, such as baseline age and sex. We adjust for covariates ${{\varvec{X}}}_{{\varvec{i}}}$:

$$\begin{array}{l}C_{it}=\beta_0+\beta_1time_{it}+\varvec{{\beta}_2^ {T}}\varvec{X}_{i}+b_{0i}+\epsilon_{it}\\b_{0i}\sim N\left(0,\sigma_b^2\right),\epsilon_{it}\sim N(0,\sigma^2)\end{array}$$

where ${\beta }_{1}$ is the slope of the composite variable over time, ${\beta }_{0}$ and ${{\varvec{\beta}}}_{2}$ are the intercept and effects of baseline covariates, respectively. ${b}_{0i}$ is the random intercept and ${\epsilon }_{it}$ is the random error.

- HR: To obtain the HRs of a time-to-event outcome (e.g., mortality) for each unit increase in the composite variable, we perform a Cox model in which we include the imputed time-varying composite variable as the main predictor, as well as baseline covariates:

$${\lambda }_{i}\left(t|{C}_{it}, {{\varvec{X}}}_{{\varvec{i}}}\right)={\lambda }_{0}(t)exp({\gamma }_{1}{C}_{it}+{{\varvec{\gamma}}}_{2}^{{\varvec{T}}}{{\varvec{X}}}_{{\varvec{i}}})$$

where ${\lambda }_{0}(t)$ is the baseline hazard function, ${\gamma }_{1}$ is log HR for each unit increase in the composite variable, ${{\varvec{\gamma}}}_{2}$ represents the effects of baseline covariates.

### Two-stage MI

**Fig. 1:**
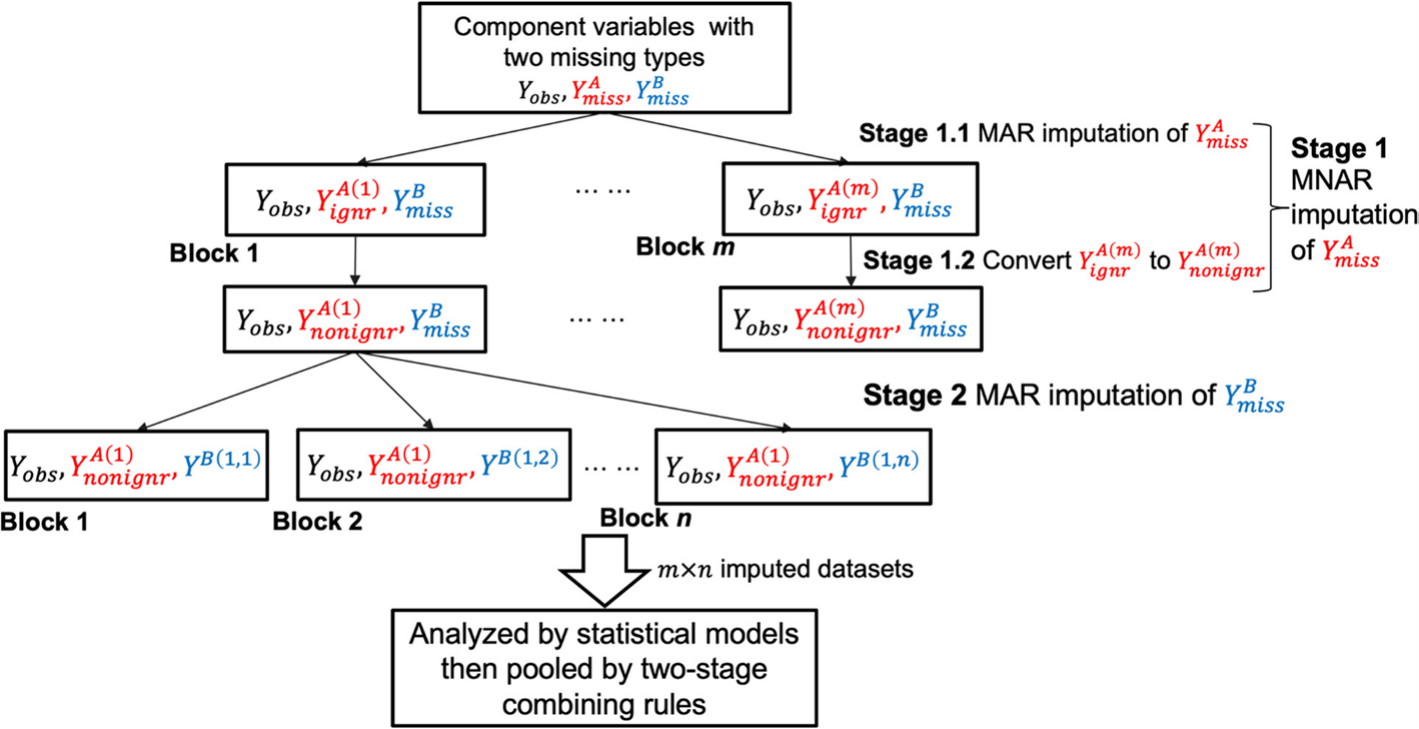
Flowchart of two-stage MI. MI, multiple imputation; MAR, missing at random; MNAR, missing not at random

We assume that data $Y$ for individual components of the composite variable can be partitioned into three parts: the observed data, ${Y}_{obs}$, data with missingness type A, ${Y}_{miss}^{A}$, and data with missingness type B, ${Y}_{miss}^{B}$. We further assume that missingness type A is MNAR and missingness type B is MAR. Note that in the FHS Offspring cohort data, we consider intermittent missingness as MAR and monotone missingness (dropout) as MNAR. If missing data is MAR or MCAR, and there is a distinction between the parameter of interest and the parameter of the missing data process, the mechanism is considered ignorable. However, if either condition is not met, the mechanism is classified as nonignorable. In this study, we use'ignorable'interchangeably with'MAR'and'MCAR,'and'non-ignorable'interchangeably with'MNAR’. The two stages in the two-stage MI framework are described as follows (Fig. 1).

#### Stage 1

There are two steps in Stage 1. First, we impute the MNAR missingness (${Y}_{miss}^{A}$) in each of the individual components at each time point for $m$ times conditional on observed data ${Y}_{obs}$ using FCS methods, assuming that the missing values are MAR:

$${Y}_{ignr}^{A\left(j\right)}\sim P\left({Y}_{miss}^{A}|{Y}_{obs}\right)$$

where $j=1,\dots , m$. $P\left({Y}_{miss}^{A}|{Y}_{obs}\right)$ is the predictive distribution of ${Y}_{miss}^{A}$ conditional on ${Y}_{obs}$. $m$ imputed datasets with imputed values ${Y}_{ignr}^{A\left(j\right)}$ are created in this step. We imputed MNAR first because it has been suggested that the nonignorable missing values should be imputed first due to certain ignorability conditions in the presence of both MAR and MNAR [16].

Second, since ${Y}_{ignr}^{A\left(j\right)}$ are the imputed values for ${Y}_{miss}^{A}$ under the assumption of MAR, we transform the ignorable imputed values ${Y}_{ignr}^{A\left(j\right)}$ into their nonignorable counterparts ${Y}_{nonignr}^{A\left(j\right)}$ using a scaling factor $k$ to account for the MNAR missingness. Adjusting ignorable imputed values assuming MAR becomes necessary if the true missing mechanism for missing data is MNAR. More details will be discussed in Sect."Imputation for Data Missing due to MNAR".

#### Stage 2

For each ${Y}_{nonignr}^{A\left(j\right)}$ from Stage 1, we draw $n$ independent values of ${Y}_{miss}^{B}$ in each of the individual components, treating the imputed values ${Y}_{nonignr}^{A\left(j\right)}$ as fixed and known:

$${Y}^{B\left(j,k\right)}\sim P\left({Y}_{miss}^{B}|{Y}_{obs}, {Y}_{nonignr}^{A\left(j\right)}\right)$$

where $k=1,\dots , n$, and $j=1,\dots , m$. $n$ imputed datasets with imputed values ${Y}^{B\left(j,k\right)}$ are created nested within each of the $m$ imputed datasets from Stage 1, that is, $m\times n$ imputed datasets are created in total. After obtaining the imputed values of all individual components at each time point, we convert them into the composite variable for each corresponding time point. Analysis models of our interest are then performed on the imputed composite variable. Since these $m\times n$ imputed datasets are not independent due to the two-stage process, the traditional MI combining rules do not apply. The two-stage combining rules have been applied, resembling the form of classical analysis of variance with the imputed datasets in Stage 1 as the blocking factor [6]. The two-stage combining rules are an extension to the traditional method of combining estimates [6]. Specifically, after imputation, we have $m\times n$ total imputed datasets, ${Y}_{imputed}^{(j,k)}=\left({Y}_{obs}, {Y}_{nonignr}^{A\left(j\right)}, {Y}^{B\left(j,k\right)}\right), j=1,\dots , m, k=1,\dots ,n$. Let $Q$ be the parameter of our interest, under the large sample assumptions, we have the following distribution:

$$({\widehat{Q}-Q})\sim N(\text{0,U})$$

where $\widehat{Q}$ is the estimate of $Q$ using the complete data, and $U$ is the variance of $\widehat{Q}$. Each of the $m\times n$ imputed datasets is used to construct the estimates for $Q$ and $U$. The overall point estimate for $Q$ is

$$\overline{Q }=\frac{1}{mn}{\sum }_{j=1}^{m}{\sum }_{k=1}^{n}{\widehat{Q}}^{(j,k)}$$

where ${\widehat{Q}}^{(j,k)}$ is the estimate of $Q$ for one imputed dataset.

The variance for $\overline{Q }$ is partitioned into three components, the first part is the estimated complete data variance

$$\overline{U }=\frac{1}{mn}{\sum }_{j=1}^{m}{\sum }_{k=1}^{n}{U}^{(j,k)}$$

where ${U}^{(j,k)}$ is the estimated variance for ${\widehat{Q}}^{(j,k)}$.

The second part is the between-block imputation variance

$$B=\frac{1}{m-1}{\sum }_{j=1}^{m}{\left({\overline{Q} }_{j.}-\overline{Q }\right)}^{2}$$

where ${\overline{Q} }_{j.}$ is the average of the ${m}^{th}$ block from Stage 1.

The third part is within-block imputation variance

$$W=\frac{1}{m}{\sum }_{j=1}^{m}\frac{1}{n-1}{\sum }_{k=1}^{n}{\left({\widehat{Q}}^{\left(j,k\right)}-{\widehat{Q}}_{j.}\right)}^{2}$$

Finally, the derived total estimated variance is

$$T=\overline{U }+\left(1+\frac{1}{m}\right)B+\left(1-\frac{1}{n}\right)W$$

To account for the finite number of imputations and for the simulation error associated with that finite number of repetitions, we have $(Q-\bar{Q})/\sqrt{T}\sim t_{\nu_*}$ and degrees of freedom ${\nu }_{*}$ has the following form

$${\nu }_{*}^{-1}=\frac{1}{m-1}{\left(\frac{\left(1+\frac{1}{m}\right)B}{T}\right)}^{2}+\frac{1}{m\left(n-1\right)}{\left(\frac{\left(1-\frac{1}{n}\right)W}{T}\right)}^{2}$$

### Imputation for data missing due to MNAR

In the second step of Stage 1 within the two-stage MI framework, we use a scaling factor $k$ to transform ignorable imputed values under the MAR assumption to nonignorable imputed values under the MNAR assumption.

For continuous individual components, we use the following formula to generate nonignorable imputed values from ignorable imputed values [2, 3]:

$$\text{nonignorable imputed} \; {Y}_{it}^{p}=k\times \text{ignorable imputed} \; {Y}_{it}^{p}$$

where ${Y}_{it}^{p}$ is the value of the $p$ th continuous component for participant $i$ at time $t$, and $k$ is a value chosen by the researcher based on prior knowledge about the relationship between nonignorable and ignorable values, which reflects the ignorability assumptions regarding the missing data. For example, $k$ = 1.2 indicates the nonignorable missing values in *Y* should be 20% larger than the ignorable counterparts, reflecting MNAR missingness. Eliciting insights from experts in the relevant field can be a helpful approach when choosing a proper *k* [8, 17]. Additionally, following up with a subset of individuals who dropped out may provide a more accurate estimate of $k$.

For binary individual components, we consider $k$ to follow the following odds ratio [9]:

$$\frac{{\pi }_{nonignr}^{p}/(1-{\pi }_{nonignr}^{p})}{{\pi }_{ignr}^{p}/(1-{\pi }_{ignr}^{p})}=k$$

where ${\pi }_{nonignr}^{p}$ and ${\pi }_{ignr}^{p}$ represent the probability of the $p$ th binary component estimated from nonignorable imputed data and ignorable imputed data, respectively. Therefore, for the binary component, $k$ represents the ratio of the odds of the binary component (e.g., smoking) estimated from nonignorable imputed data as compared to the odds estimated from the corresponding ignorable imputed data. Taking natural logarithm on both sides, we have

$$logit\left({\pi }_{nonignr}^{p}\right)=\text{log}\left(k\right)+logit({\pi }_{ignr}^{p})$$

Therefore, we first use a logistic regression model to obtain $logit({\pi }_{ignr}^{p})$ for the binary variable imputed from FCS methods in the first step of Stage 1, then we apply the scaling factor $k$ to obtain the probability of their nonignorable imputed counterparts ${\pi }_{nonignr}^{p}$. The imputed binary variable based on the MNAR assumption is generated from a Bernoulli distribution with probability ${\pi }_{nonignr}^{p}$.

### FCS imputation methods

“FCS methods are employed in both stages of the two-stage MI framework to generate imputed values assuming MAR. FCS addresses multivariate missing data by imputing each variable individually in a sequential manner. This approach involves defining an imputation model for each variable with missing data and iteratively generating imputations for them [18]. FCS is originally introduced to impute missing values in cross-sectional data. Subsequently, it is extended to handle missing values in longitudinal data collected at regular intervals, treating the repeated measurements as distinct variables in the longitudinal setting [19]. Within the FCS algorithm, we evaluate two approaches for imputing the continuous components that incorporate parameter uncertainty: Bayesian linear regression and linear regression with bootstrap. The former draws parameters from posterior distributions, while the latter resamples the data using bootstrap to estimate parameters. While both methods yield comparable results, we use the bootstrap method for our analysis due to its computational efficiency with our large dataset. For the binary component, we use an approximate Bayesian imputation approach with logistic regression.

Using the previously described approaches for continuous and binary components, we implement and compare three versions of FCS with different model structures, including All FCS (AFCS), Cross-sectional FCS (XFCS), and Longitudinal FCS (LFCS). Suppose that ${Y}_{it}^{p}$ is the value of component $p$ at time $t$ ($t=\text{1,2},\dots , T, p=1,\dots , P)$, these three versions of FCS are specified as follows:

- XFCS imputes ${Y}_{it}^{p}$ using other components at time $t$, ${Y}_{it}^{-p}$, while not incorporating longitudinal information.
- LFCS only considers longitudinal information of the variable being imputed, and it imputes ${Y}_{it}^{p}$ using component $p$ at all other time points, ${Y}_{i,-t}^{p}$.
- AFCS considers both longitudinal and cross-sectional information in LFCS and XFCS.

## Simulation study design

In this section, we performed a simulation study to address the three questions raised in Sect."Data Setup and Analysis Models of Interest"**.**

### Generation of full data

We simulated five individual components ${Y}_{it}^{p} (p=\text{1,2}\dots ,5)$ in the composite variable, including one binary component and four continuous components, across five time points for 3700 subjects. These five components mimicked those in Composite- 5, where higher values for individual components and a lower overall composite score indicated poorer cardiovascular health. At each time point, the five components were simulated from a multivariate normal distribution using metrics (e.g., mean, standard deviation [SD], and correlation) derived from the FHS longitudinal data, both to reflect real data and to allow for generalization. Specifically, the means of these simulated components exhibited a non-decreasing trend over time. We specified moderate to high within-component correlations across exams and low between-component correlations. The time points were evenly spaced every five years. In addition, we simulated two baseline covariates, one binary and one continuous, designed to mimic sex and baseline age from the FHS data. These two variables were generated along with the components using a multivariate normal distribution. Binary variables were initially simulated as continuous values and then converted to binary outcomes using a quantile-based threshold that ensures the positive outcome probability matches the target rate. Parameter values for means, SDs, and correlation structure were presented in Supplementary Material 1: Table S2, Table S3, with correlation heatmap shown in Supplementary Material 1: Figure S1.

### Generation of missing data

To mimic the FHS data, we generated MAR and MNAR missingness of the data values. To do this, we divided the generated data into two equal parts (i.e., the MAR group and the MNAR group), with each part subject to only one of the missing mechanisms.

For the MAR group, we introduced missingness in individual components and non-attendance (i.e., missingness in all components) from the second to the fifth time points. Specifically, we used logistic regressions to predict the missingness of two components at the current time point based on their values at baseline as well as observed baseline covariates. As for MAR non-attendance, we used the composite variable at baseline to predict non-attendance at the current time point using logistic regressions. The logistic regressions utilized coefficients estimated from the FHS Offspring cohort data to calculate predicted probabilities of missingness. Missing indicators were then randomly assigned using Bernoulli distributions based on these predicted probabilities. The logistic regression coefficients were provided in Supplementary Material 1: Table S4.

For the MNAR group, we generated non-attendance only, because a participant’s changing health condition (i.e., improving or deteriorating) would more likely lead to complete withdrawal from an exam rather than missing only individual components. In Table 1, we created three scenarios of MNAR non-attendance. In Scenario 1, we set all components for a participant to missing from the second to the fifth time point with an 80% probability if their composite variable fell within the lower percentiles (bottom 10 th, 15 th, 20 th, and 25 th at each respective time point), reflecting a higher likelihood of non-attendance due to declining health. In Scenario 2, we assumed that healthier participants, whose composite variable fell within the upper percentiles, were more likely to view health exams as redundant and therefore failed to attend them. In Scenario 3, we created non-attendance for participants with the composite variable values within both upper and lower percentiles on the assumption that both Scenario 1 and Scenario 2 were possible.

**Table 1:** Scenarios of missingness in the MNAR group

Scenarios	Steps
1	(i) Set all individual components for a participant to missing at an exam if the participant’s composite variable fell within the **lower** percentiles for that exam (ii) Removed all observations occurring after the simulated death time for each participant^a^
2	(i) Set all individual components for a participant to missing at an exam if the participant’s composite variable fell within the **upper** percentiles for that exam (ii) Removed all observations occurring after the simulated death time for each participant^a^
3	(i) Set all individual components for a participant to missing at an exam if the participant’s composite variable fell within **both upper or lower percentiles** for that exam (ii) Removed all observations occurring after the simulated death time for each participant^a^

*MNAR* missing not at random

^a^Both Step (i) and Step (ii) were implemented for the Cox model to address Question (iii), while only Step (i) was employed when evaluating the mean and slope of the composite variable to answer Question (i) and Question (ii)

The dataset with missing values generated in this way was used to address the first two questions regarding estimating the average of the composite variable at each time point and estimating the slope of the composite variable over time. Table 2 summarized the missing percentage for each missing mechanism in the simulated data.

**Table 2:** Missing percentage in the simulated data

	**Time Point 2**	**Time Point 3**	**Time Point 4**	**Time point 5**
Two individual components	5	3	8	9
MAR Non-attendance	5	4	8	11
MNAR Non-attendance	10	12.5	15	17.5

### Generation of event times

To evaluate the Cox model from Question (iii), we further simulated event times (e.g., time to death) and removed data after the event based on the dataset simulated in Sect."Generation of Missing Data". Therefore, the dataset used to evaluate the Cox model incorporated missing data generated from Sect."Generation of Missing Data". Specifically, we simulated time to death for each 5-year interval based on the composite variable using piecewise exponential models, and then we removed the data after the simulated death time for each participant and treated these removed data as missing. Detailed steps were summarized in Supplementary Material 1: Method S1.

Note that when incorporating missingness due to death into each of the three scenarios in Table 1, each simulated dataset included missing data with one MAR mechanism and at least two MNAR mechanisms. Specifically, in Scenario 1, one of the two MNAR mechanisms caused missing data based on the assumption that participants were more likely to miss an exam due to their worsening health conditions (i.e., lower composite variable). The other MNAR mechanism led to missing data due to death, which was also associated with the participants’ deteriorating health conditions. In Scenario 2, the two MNAR mechanisms in Scenario 2 were contradictory: one led to missing data associated with better cardiovascular health, while the other caused missing data due to death, reflecting worse cardiovascular health. In Scenario 3, the datasets included three distinct MNAR mechanisms: two associated with worse cardiovascular health and one with better cardiovascular health.

### Imputation of the composite variable

We imputed the missing values in each individual component of the composite variable within the two-stage MI framework (Fig. 1). We assumed we had prior knowledge of which participants belonged to the MAR group and which were in the MNAR group. Specifically, we imputed missing values in individual components using multiple ignorability assumptions ($k=0.8, 0.9, 1, 1.1, \text{and} \; 1.2)$ in Stage 1 for the MNAR group. Next, we treated the imputed values in Stage 1 as fixed and known, and imputed missing values in the MAR group in Stage 2. After the imputation of individual components, we constructed the composite variable based on the criteria described in Supplementary Material 1: Table S1.

We tried a few different numbers of imputation in two stages (e.g., *m* = 5,10,15 and *n* = 5,10,15), and all the results were consistently similar. Therefore, we chose *m* = *n* = 5 in both stages. We employed three FCS methods (i.e., AFCS, LFCS, XFCS) in both stages. Table 3 illustrated the imputation of a continuous component representing BMI at the third time point, along with the predictors used in the imputation models. Note that for the analysis of Cox model, we treated data after death as missing and imputed them even though they were not used in the Cox model. There were mainly three reasons: first, the FCS methods treated each component at each time point as a distinct variable, imputing missing values regardless of the missing mechanism. As a result, it was not feasible to selectively impute only data missing due to MAR and MNAR as discussed in Sect."Generation of Missing Data"while leaving missing data due to death unimputed; second, all components at all time points needed to be fixed and known before entering Stage 2 of the two-stage MI framework, which required imputing missing data due to death; third, our approach can be generalized to non-terminal events other than death, such as dementia. In these scenarios, data following non-terminal events can be considered as missing data.

**Table 3:** Predictors in the imputation models for the imputation of BMI at time point 3

FCS methods	Predictors in the imputation models for $BM{I}_{3}$
AFCS	$BM{I}_{1}, BM{I}_{2}, BM{I}_{4}, BM{I}_{5},$ $SB{P}_{1}, SB{P}_{2}, SB{P}_{3}, SB{P}_{4}, SB{P}_{5},$ $DB{P}_{1}, DB{P}_{2}, DB{P}_{3}, DB{P}_{4}, DB{P}_{5},$ $T{C}_{1}, T{C}_{2}, T{C}_{3}, T{C}_{4}, T{C}_{5},$ $B{G}_{1}, B{G}_{2}, B{G}_{3}, B{G}_{4}, B{G}_{5},$ $SM{K}_{1}, SM{K}_{2}, SM{K}_{3}, SM{K}_{4}, SM{K}_{5},$ baseline age, sex, death indicator^a^, $H_0\left(T\right)$^a^
LFCS	$BM{I}_{1}, BM{I}_{2}, BM{I}_{4}, BM{I}_{5},$ baseline age, sex, death indicator^a^, $H_0\left(T\right)$^a^
XFCS	$BM{I}_{3}, SB{P}_{3}, DB{P}_{3}, T{C}_{3}, B{G}_{3},$ baseline age, sex, death indicator^a^, $H_0\left(T\right)$^a^

$BM{I}_{j}$ body mass index at time point$j$; $SB{P}_{j}$ systolic blood pressure at time point$j$; $DB{P}_{j}$ diastolic blood pressure at time point$j$; $T{C}_{j}$ total cholesterol at time point$j$; $B{G}_{j}$ fasting blood glucose at time point$j$; $, SM{K}_{j}$ smoking status at time point$j$;${H}_{0}(T)$, the estimated cumulative null hazard for mortality

^a^Only included in imputation models if our analysis model is the Cox model

Table 4 showed different ignorability assumptions ($k$ values) in two-stage MI for the relationship between ignorable imputed data and their nonignorable conterparts. Table 5 further provided interpretations for each combination of scenarios of missingness in Table 1 and ignorability assumptions in two-stage MI. When missingness due to death was not considered, for Scenario 1, we assumed that non-attendees with MNAR data had worse cardiovascular health than others. Therefore, $k$ values of 1.1 and 1.2 were considered the appropriately specified MNAR assumption since this multiplier $k$ increased the imputed values of individual components (e.g., higher SBP level), leading to smaller imputed values of the composite variable that reflected health deterioration. Similarly, in Scenario 2, $k$ values of 0.8 and 0.9 were considered appropriate since both the missing data scenario and the ignorability assumption in two-stage MI assumed missingness due to better health conditions. In Scenario 3, none of the $k$ values were considered appropriate. When considering missingness due to death, for Scenario 1, since both MNAR mechanisms postulated that missing data were associated with declining health (i.e., lower composite variable), $k$ values of 1.1 and 1.2 were considered appropriate. In Scenarios 2 and 3, none of the $k$ values were appropriate, since the MNAR mechanisms were contradictory, resulting in missingness related to both worse and better cardiovascular health. Note that even though the imputed data due to death was not utilized in the Cox model, they still influenced the imputed data under MAR in Stage 2 of the two-stage MI framework.

**Table 4:** Ignorability assumptions in two-stage MI

${\varvec{k}}$ for continuous components	${\varvec{k}}$ for binary component	Assumption	Interpretation in the simulated data
1	1	MAR missingness	MNAR participants have similar cardiovascular health as compared to other participants
		nonignorable imputed values for individual components = ignorable counterparts;	
		nonignorable imputed composite variable = ignorable counterparts	
1.2 or 1.1	2	MNAR missingness	MNAR participants have worse cardiovascular health than other participants
		nonignorable imputed values for individual components > ignorable counterparts	
		nonignorable imputed composite variable $\le$ ignorable counterparts	
0.9 or 0.8	0.5	MNAR missingness	MNAR participants have better cardiovascular health than other participants
		nonignorable imputed values for individual components < ignorable counterparts	
		nonignorable imputed composite variable $\ge$ ignorable counterparts	

*MAR* missing at random, *MNAR* missing not at random

**Table 5:** Different ignorability assumptions ($k$= 0.8, 0.9, 1, 1.1, 1.2) for three scenarios of missingness

Scenarios of missingness	Ignorability assumptions in two-stage MI		
	$k$= 1 (continuous)	$k$= 1.1 and 1.2 (continuous)	$k$= 0.8 and 0.9 (continuous)
	$k$= 1 (binary)	$k$= 2 (binary)	$k$= 0.5 (binary)
1	MAR	Appropriately specified MNAR	Misspecified MNAR
2	MAR	Misspecified MNAR	Appropriately specified MNAR^a^
3	MAR	Misspecified MNAR	Misspecified MNAR

Two-stage MI, two-stage multiple imputation; MAR, missing at random; MNAR, missing not at random

^a^Misspecified MNAR when incorporating missingness due to death for the analysis of hazard ratios in the Cox model

We also employed complete case (CC) analysis and Last Observation Carried Forward (LOCF) as two comparison methods. In CC analysis, if a participant missed any individual component at a certain time point, all components were removed for that participant at that time point. For LOCF, we filled the missing value of a component at a time point using the value of the same component from the previous time point.

### Evaluation of two-stage MI

After imputation, we investigated the means, slopes across time points, and HRs of the imputed composite variable, as described in Sect."Data Setup and Analysis Models of Interest". We obtained the point estimates and inferences for two-stage MI using the two-stage combining rules. We used percent bias and coverage probability to examine the performance of the methods. Data were generated, imputed, and analyzed for 500 times. To obtain the true parameter values for the means and slopes of the composite variable over time, we simulated a full dataset with 1,000,000 participants as the underlying population using the mean, SD, and correlation structure described in Sect."Generation of Full Data". The mean of the composite variable derived from this full dataset at each time point served as the true mean. Similarly, the estimated slope of the composite variable served as the true value for slope. The pre-specified log HR of mortality, which was used to simulate time to death, served as the true value for log HR. All statistical analyses were conducted using R (version 4.1.2; R Foundation for Statistical Computing). FCS methods were implemented using R package “mice” [20]. The code for the two-stage combining rules was modified from Siddique et al. (2012) [8].

## Results of simulation study

In this section, we compared the simulation results for two-stage MI with multiple ignorability assumptions as well as CC analysis and LOCF.

### Results for means and slopes

**Fig. 2:**
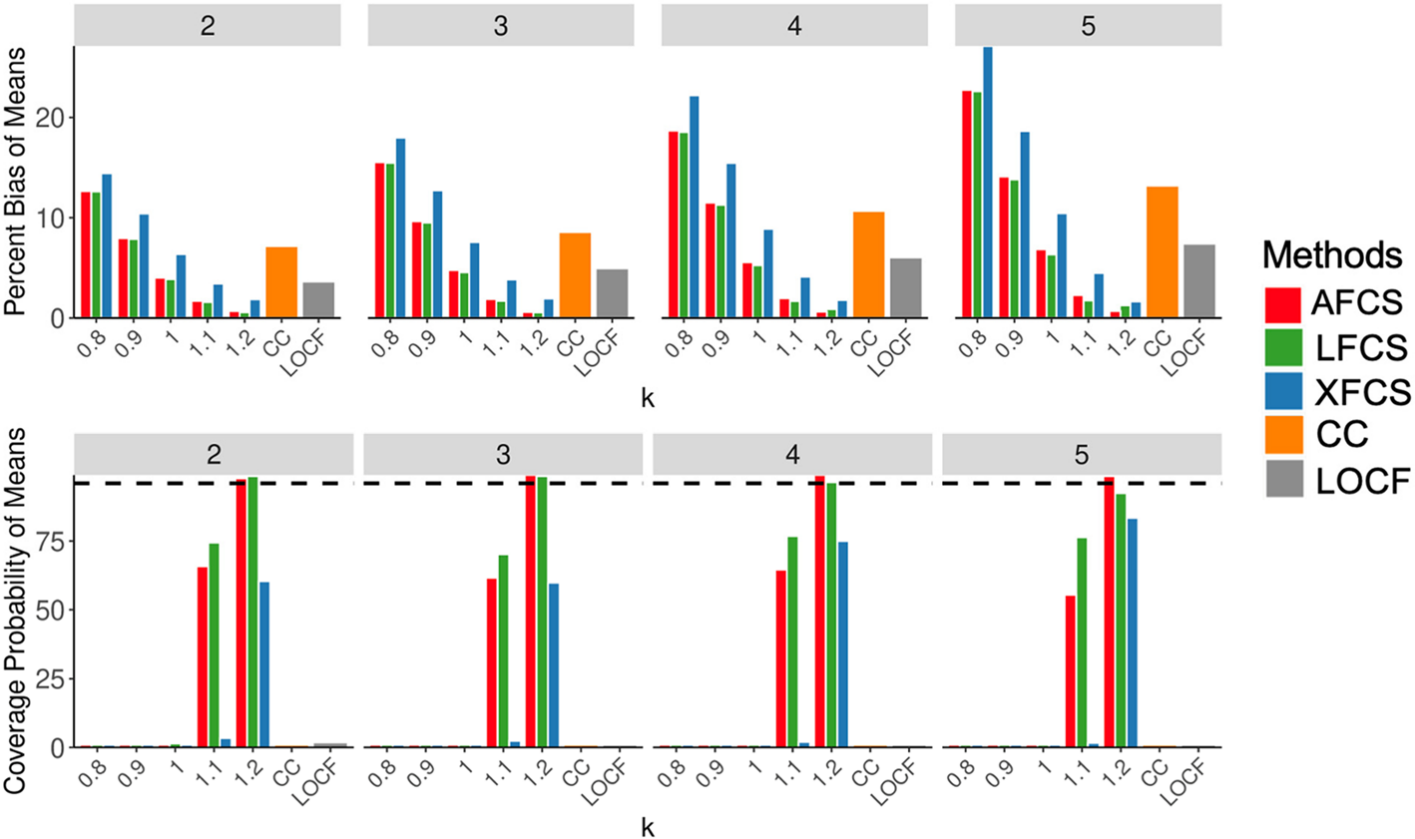
Simulation results for the mean of the composite variable in Scenario 1. The x-axis in each panel denotes various ignorability assumptions in two-stage MI. The top panel displays the percent bias of means (%), while the bottom panel illustrates the coverage probability of means (%). Four subpanels in each of the two panels represent four time points. The dashed line in the lower panel is the 95% coverage probability. $k$= 0.8 and 0.9 represent the misspecified MNAR assumption, $k$ = 1 represents the MAR assumption, $k$ = 1.1 and 1.2 represent the appropriately specified MNAR assumption. MAR, missing at random; MNAR, missing not at random; AFCS, all fully conditional specification; CC, complete case analysis; LFCS, longitudinal fully conditional specification; XFCS, cross-sectional fully conditional specification; LOCF, last observation carried forward

The top panel in Fig. 2 showed us the percent bias in Scenario 1. As the missing percentage increased from 10% at the second time point to 25% at the fifth time point, the percent bias also increased. The appropriately specified MNAR assumption ($k$ = 1.1, 1.2) produced the smallest percent bias, ranging from 0.5% to 4%, across all imputation methods while the misspecified MNAR assumption ($k$ = 0.8, 0.9) and CC analysis yielded more biased estimates (highest percent bias: 27%). Both AFCS and LFCS imputation methods that considered longitudinal information were able to recover data with similar unbiased estimates (percent bias: 0.5% to 2%) under the appropriately specified MNAR assumption, outperforming XFCS. LOCF and the MAR assumption ($k$ = 1) produced similar percent bias which fell between the appropriately specified and misspecified MNAR assumptions. The bottom panel in Fig. 2 showed coverage probabilities. CC analysis, LOCF, and the misspecified MNAR and MAR assumptions were consistently zero for each FCS method at each time point. In contrast, the appropriately specified MNAR assumption resulted in more accurate coverage probabilities, with LFCS or AFCS achieving the highest coverage across all time points, reaching a peak of 98%.

In Scenario 2 (Supplementary Material 1: Figure S2), the appropriately specified MNAR assumption ($$k=0.8, 0.9)$$ produced the most accurate estimates using AFCS and LFCS. In Scenario 3 (Supplementary Material 1: Figure S3), where none of the $$k$$ values were appropriately specified for ignorability assumptions, the MAR assumption ($$k$$ = 1), CC analysis, and LOCF performed similarly and yielded unbiased estimates, outperforming other ignorability assumptions using two-stage MI. Although the MAR assumption failed to accurately recover both percentiles in the composite variable, the biases from both percentiles were offset. Similarly, CC analysis and LOCF produced relatively unbiased estimates, as ignoring both percentiles had little impact on the means of the composite variable.

**Fig. 3:**
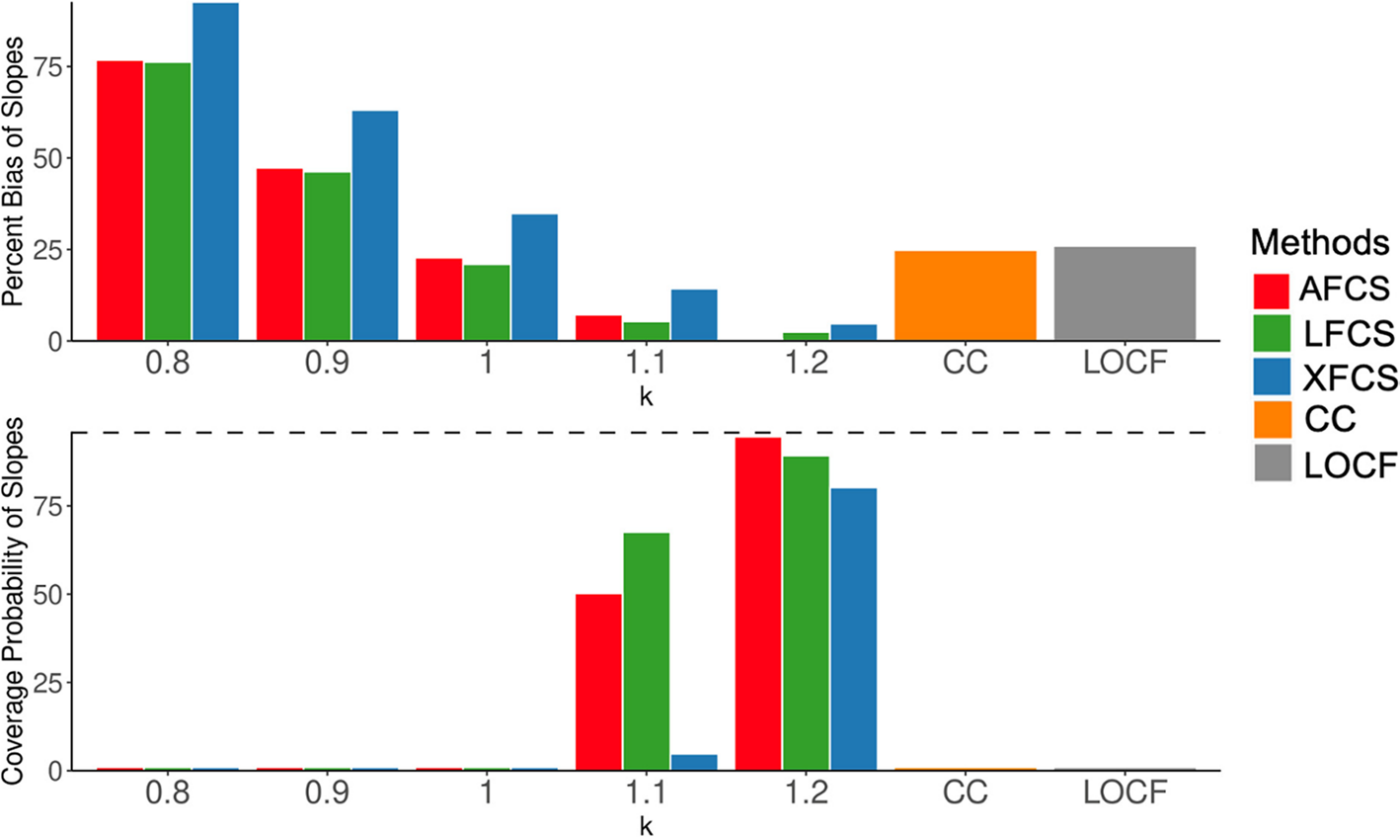
Simulation results for the slope of the composite variable in Scenario 1. The x-axis in each panel denotes various ignorability assumptions in two-stage MI. The top panel displays the percent bias of slope (%), while the bottom panel illustrates the coverage probability of slope (%). The dashed line in the lower panel is the 95% coverage probability. $k$= 0.8 and 0.9 represent the misspecified MNAR assumption, $k$ = 1 represents the MAR assumption, $k$ = 1.1 and 1.2 represent the appropriately specified MNAR assumption. MAR, missing at random; MNAR, missing not at random; AFCS, all fully conditional specification; CC, complete case analysis; LFCS, longitudinal fully conditional specification; XFCS, cross-sectional fully conditional specification; LOCF, last observation carried forward

Most of the findings drawn from the means of the imputed composite variable can also be applied to the slopes of the composite variable over time. In Scenarios 1 and 2 (Fig. 3 and Supplementary Material 1: Figure S4), the appropriately speficied MNAR assumption in two-stage MI yielded the best results and the MAR assumption produced intermediate results between appropriate and misspecified assumptions. AFCS and LFCS outperformed XFCS in most scenarios and surpassed CC analysis and LOCF when the MNAR assumption was correctly specified. In Scenario 3 (Supplementary Material 1: Figure S5), the MAR assumption produced the most accurate estimates.

### Results for mortality HR

**Fig. 4:**
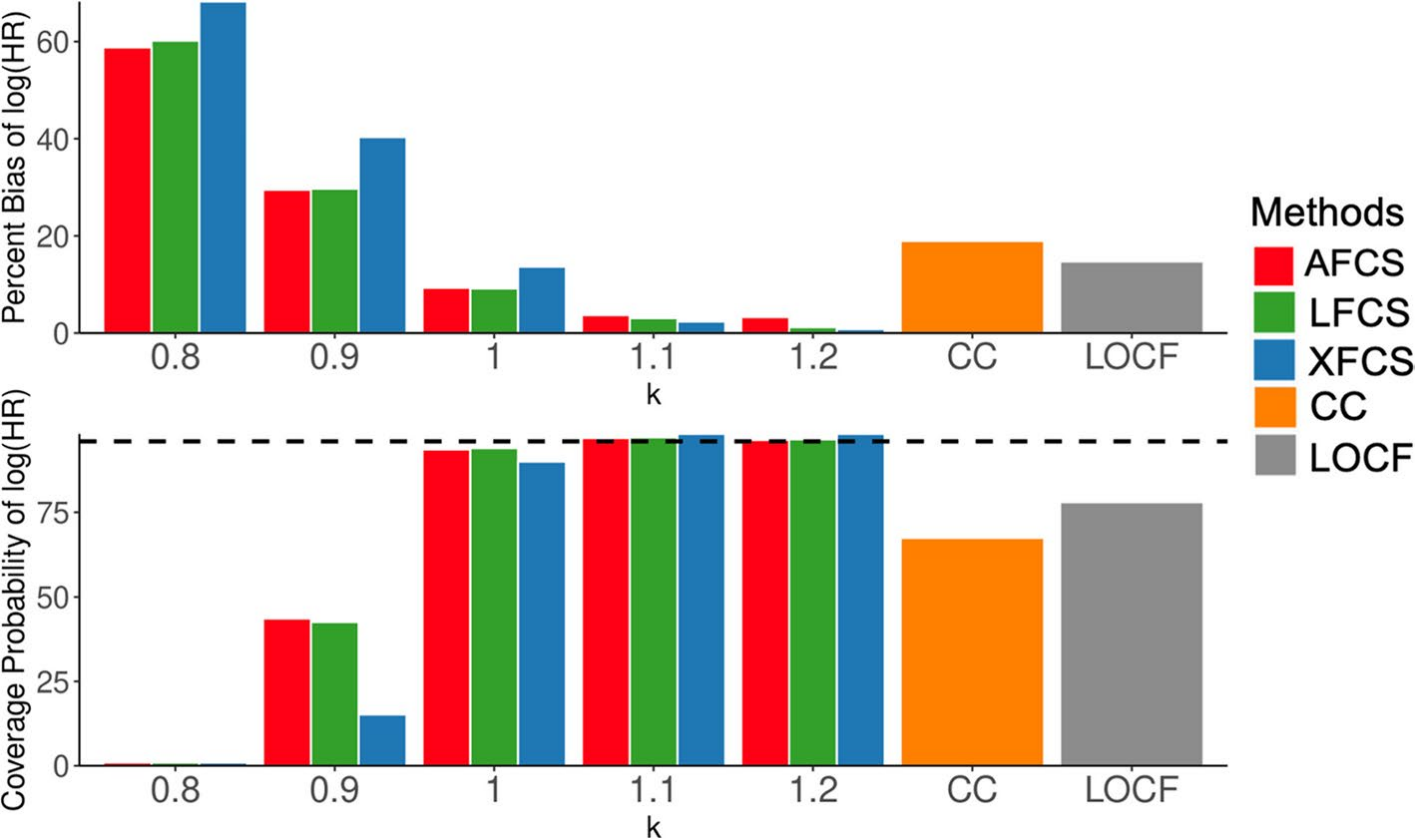
Simulation results for log HR of the composite variable in Scenario 1. The x-axis in each panel denotes various ignorability assumptions in two-stage MI. The top panel displays the percent bias of log HR (%), while the bottom panel illustrates the coverage probability of log HR (%). The dashed line in the lower panel is the 95% coverage probability. $k$= 0.8 and 0.9 represent the misspecified MNAR assumption, $k$ = 1 represents the MAR assumption, $k$ = 1.1 and 1.2 represent the appropriately specified MNAR assumption. MAR, missing at random; MNAR, missing not at random; AFCS, all fully conditional specification; CC, complete case analysis; LFCS, longitudinal fully conditional specification; XFCS, cross-sectional fully conditional specification; LOCF, last observation carried forward

In this section, we showed the results for log HR of mortality for each unit increase in the composite variable. In Scenario 1 (Fig. 4), the appropriately specified MNAR assumption ($k=1.1$, $1.2$) produced unbiased log HR (percent bias: 0.3% to 3%) and the largest coverage probabilities (range: 95% to 97%) for all three FCS methods. Misspecified MNAR assumption ($k=0.8$, 0.9) yielded biased log HR that ranged from 29 to 68% and small coverage probabilities from 0 to 43%. When missing data were considered MAR ($k=1$), the percent bias for log HR was around 10% and the coverage probabilities were between 90 and 93%. Both appropriately specified MNAR and MAR assumptions showed improvement over CC analysis and LOCF. All three FCS methods showed unbiased results under the appropriately specified MNAR assumption, while AFCS and LFCS performed better than XFCS under other ignorability assumptions.

In Scenario 2 (Supplementary Material 1: Figure S6), where two MNAR mechanisms were contradictory in the data, the MAR assumption ($k=1$) outperformed all other ignorability assumptions that treated two different MNAR mechanisms in the same manner. AFCS and LFCS under the MAR assumption also outperformed LOCF and CC analysis. In Scenario 3 (Supplementary Material 1: Figure S7) which involved three MNAR mechanisms, the MAR assumption and the MNAR assumptions with $k=1.1$ and 1.2 performed similarly, surpassing the MNAR assumptions with $k=0.8$ and 0.9. The misspecified MNAR assumptions with $k=1.1$ and 1.2 performed well due to their consistency with two of the three MNAR mechanisms in the simulated data, which indicated an association between lower composite variable and larger probability of missing an exam. AFCS and LFCS showed improvement over XFCS under all ignorability assumptions.

## Real data application

In this section, we demonstrated the application of two-stage MI to the FHS Offspring cohort data [13]. We mainly focused on the period between exam 5 to exam 9, because the decline in cardiovascular health was more pronounced and showed more variability after exam 5 (mean age 55 years) compared to the first four exams. Besides, most of death cases occurred in older participants after exam 5. We further restricted the data to participants who were measured with all five components of Composite- 5 at exam 5 (N = 3706), which served as the baseline of our study. We first applied the two-stage MI framework to assess the means and slopes of Composite- 5 across exam 5 to exam 9, then we assessed the log HR of all-cause mortality for each unit increase in Composite- 5 using Cox proportional hazards models.

### Methods

The subjects in the FHS Offspring cohort can be partitioned into three groups: those with complete data, those with intermittent missing data, including missing values in individual components and all components, and those with monotone missing data (dropout). We imposed the MAR and MNAR mechanisms on the two groups with missingness. Intermittent missingness was assumed to be MAR [21]. We further assumed monotone missingness (dropout) to be MNAR. Note that if participants missed the final exam, they were classified as dropout, since the nonattendance was more likely to be attributed to worse health conditions due to older age at later exams. We also conducted sensitivity analyses to evaluate the impact of treating these participants as MAR.

To impute components in Composite- 5, we followed the same steps described in Sect."Methods"and Sect."Simulation Study Design". We first evaluated whether two-stage MI preserved the mean of Composite- 5 at each exam and its temporal trend over time. We then focused on whether Composite- 5 predicted all-cause mortality using Cox models. To compare Cox models with different imputation methods, several Cox models were considered, including 2Stage, CC-AVAIL, and CC-exam5 to CC-exam9 (Table 6). Model 2Stage included the imputed longitudinal Composite- 5 as a time-varying predictor, CC-AVAIL utilized Composite- 5 that was constructed with all available information across exams. Composite- 5 was also imputed using LOCF, and the other models used the complete data at each specific exam after excluding participants with missing components.

**Table 6:** Cox models considered for predicting all-cause mortality

Model name	Predictor in the Cox model^a^	Sample size
2Stage ($k$^b^)	time-dependent Composite- 5 imputed within the two-stage MI framework	3706
CC-AVAIL	time-dependent Composite- 5 constructed with all available data	3706
LOCF	time-dependent Composite- 5 imputed using LOCF	3706
CC-exam5	Composite- 5 at exam 5	3706
CC-exam6	Composite- 5 at exam 6	3231
CC-exam7	Composite- 5 at exam 7	3033
CC-exam8	Composite- 5 at exam 8	2600
CC-exam9	Composite- 5 at exam 9	2057

^a^All models were adjusted for baseline age and sex

^b^ $$k$$ = 0.8, 0.9, 1, 1.1, or 1.2

### Results for means and slopes

**Fig. 5:**
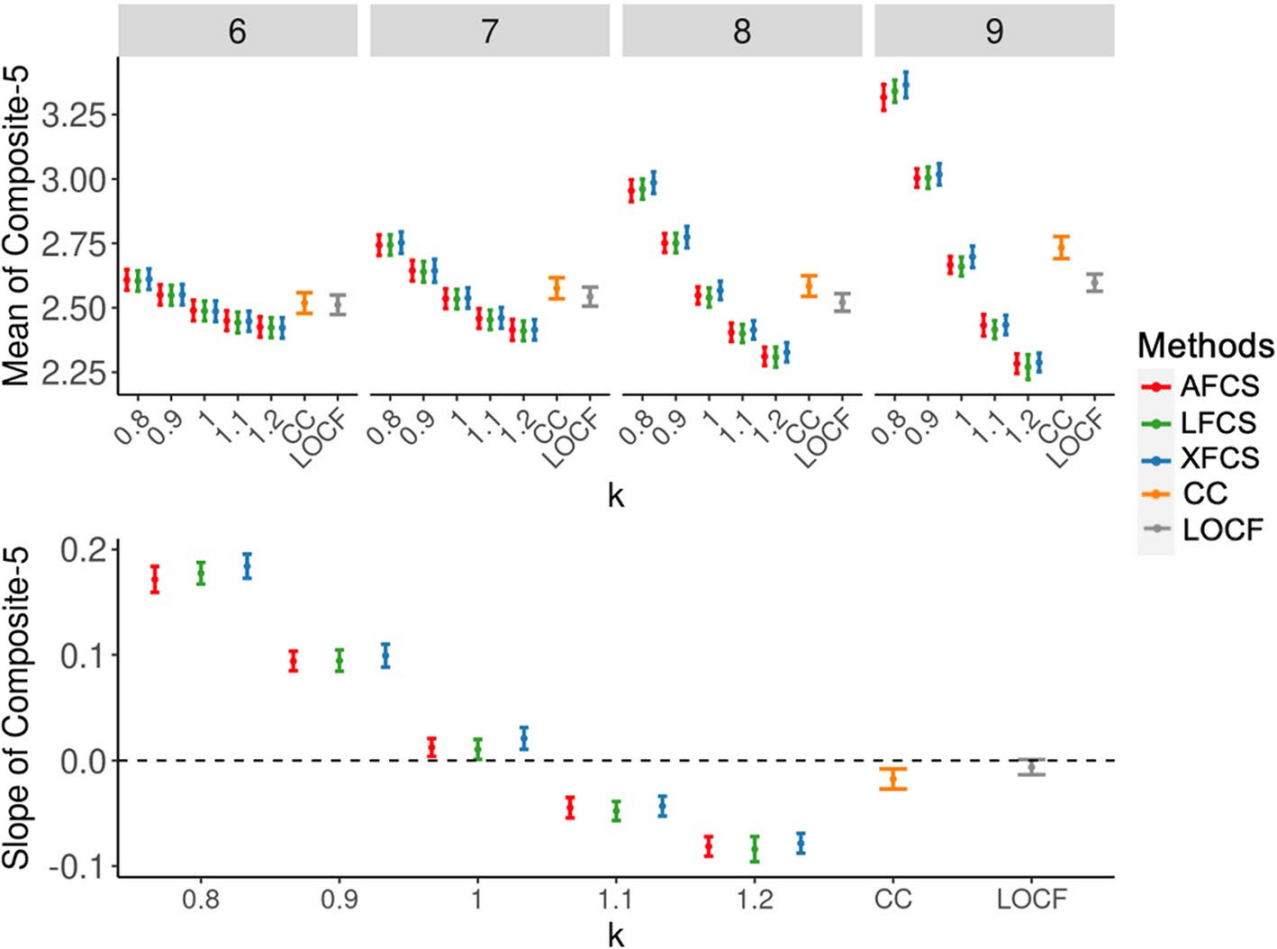
Means and slopes of Composite- 5 in the FHS Offspring cohort. In the top panel, the y-axis represents the mean values of Composite- 5, and the x-axis represents different ignorability assumptions in two-stage MI. Each bar denotes the point estimate and its 95% CI for the mean value of Composite- 5. Four subpanels illustrate four exams. In the bottom panel, the y-axis represents the slope values of Composite- 5 across exams in the linear mixed effects models, and the x-axis represents different ignorability assumptions in two-stage MI. Each bar denotes the point estimate and its 95% CI for the slope value of Composite- 5. MI, multiple imputation; AFCS, all fully conditional specification; LFCS, longitudinal fully conditional specification; XFCS, cross-sectional fully conditional specification; CC, complete case analysis; LOCF, last observation carried forward; CI, confidence interval

In the FHS Offspring cohort data, both intermittent and monotone missing patterns were observed (Supplementary Material 1: Figure S8), and monotone missingness was the most common pattern (*n* = 1377; 37%). For any particular exam and ignorability assumptions, all three FCS methods performed similarly in terms of the mean and slope of Composite- 5 (Fig. 5). In the FHS health exams, one would expect that the Composite- 5 values to be lower among those who dropped out conditioning on the observed information, due to poorer cardiovascular health compared to the attendees. Therefore, the MAR assumption ($k$= 1), CC analysis, and LOCF may introduce a survival bias, resulting in a distortion in the mean scores of Composite- 5 with a tendency towards higher values (range:2.5 to 2.8). To account for the potential MNAR missingness, we employed $k$ = 1.1 and 1.2, assuming those who dropped out had poorer cardiovascular health than the participants who remained in the study (Table 4). The estimated means of Composite- 5 for $k$= 1.1 and 1.2 were lower (range: 2.25 to 2.5) than other ignorability assumptions as well as CC analysis and LOCF. This adjustment helped mitigate the potential survival bias resulting from MNAR. In addition, as the rate of dropout increased from exam 7 to exam 9, the disparities between different ignorability assumptions became more pronounced.

We further investigated the slopes of Composite- 5 over the exams in the linear mixed models adjusting for baseline age and sex (bottom panel in Fig. 5). The estimates from XFCS were slightly higher than the other two FCS methods. Using LOCF and CC analysis, Composite- 5 exhibited an average decrease of 0 to 0.02 units between each successive exam, while the slope of Composite- 5 assuming MAR displayed a shift in direction, as it considered comparable health statuses of participants who left the study and those who remained. When $k$ = 1.1 and 1.2 under the assumption of MNAR, the estimated Composite- 5 showed a decrease over time, with the most pronounced change of − 0.08 observed for LFCS. This indicated that the average cardiovascular health declined over time under the assumption that the participants who had poorer health statuses were more likely to drop out. On the contrary, Composite- 5 exhibited an increasing trend over time for $k$= 0.8 and 0.9, with the slopes ranging from 0.1 to 0.18.

### Results for mortality HR

**Fig. 6:**
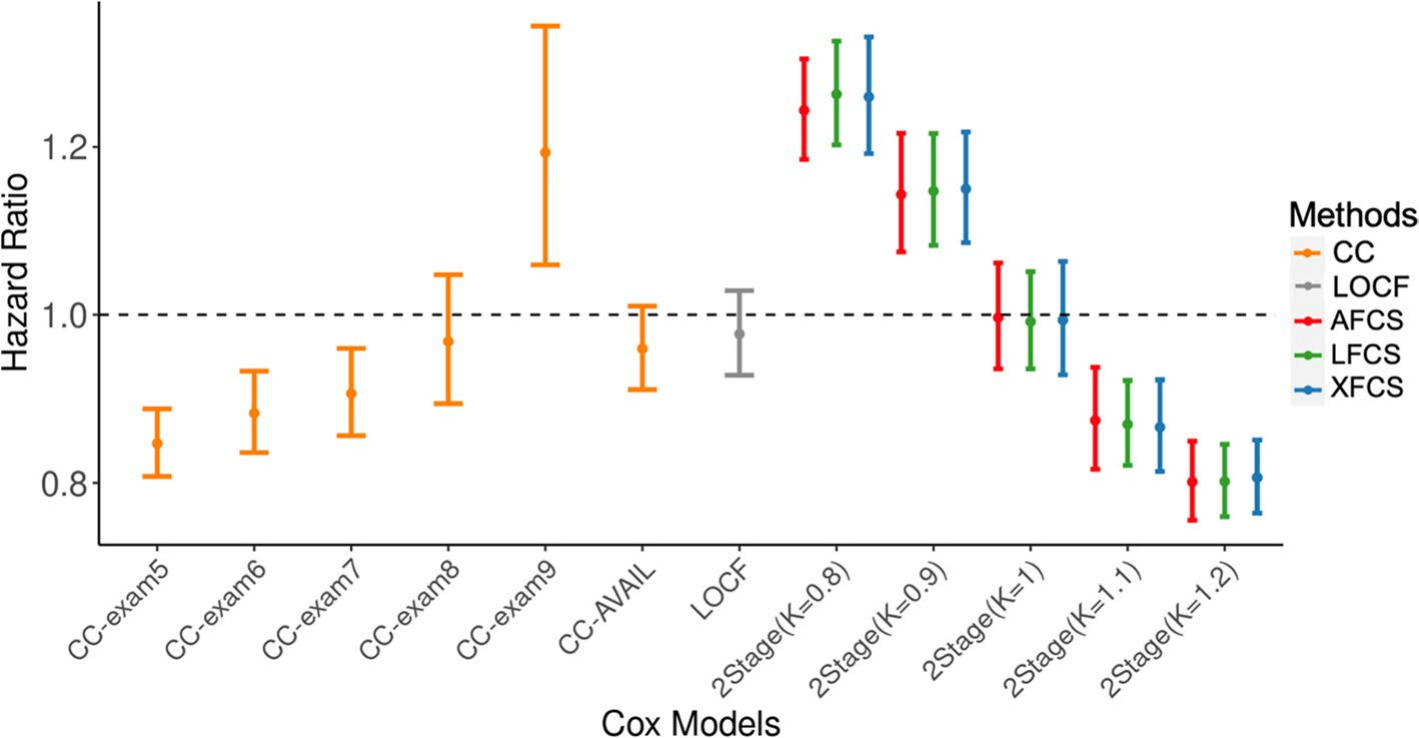
HRs in the FHS Offspring cohort. The y-axis represents the HRs of Composite- 5 in Cox models, and the x-axis represents different Cox models in Table 6 Each bar denotes the point estimate and its 95% CI for the HR of death for each unit increase in Composite- 5. HR, hazard ratio; AFCS, all fully conditional specification; LFCS, longitudinal fully conditional specification; XFCS, cross-sectional fully conditional specification; CC, complete case analysis; LOCF, last observation carried forward; CI, confidence interval

Among the FHS participants, 3706 participants had complete data at exam 5 and were followed up until the end of 2019, with a median follow-up of 26 years. Of them, 1465 (~ 40%) died during follow-up (median 18 years). Figure 6 showed the HRs of all-cause mortality corresponding to 1-unit higher level of Composite- 5, adjusting for baseline age and sex. CC-AVAIL that used all available data across exams and LOCF found no association between time-dependent Composite- 5 and all-cause mortality. However, for the other models with no imputation, the estimated HRs showed a consistently increasing trend in response to 1-unit higher level in Composite- 5 from exam 5 to exam 9. Specifically, CC-exam5, CC-exam6, and CC-exam7 showed significantly reduced hazard of all-cause mortality (HR range: 0.85 $-$ 0.91) with relatively narrower 95% CIs. However, the estimated HR for CC-exam8 became nonsignificant, and it turned harmful in CC-exam9 (HR = 1.19; 95% CI 1.06 $-$ 1.34). The shift in the direction of HR was likely attributed to the survival bias, i.e., the dropout rate of participants with worsening health conditions increased over time.

Next, we focused on Cox models with imputed Composite- 5 (Fig. 6). Under various ignorability assumptions in two-stage MI, all three FCS methods yielded comparable point estimates for HR and 95% CI. 2Stage ($k$= 1) under the MAR assumption generated nonsignificant association, aligning with the results in CC-AVAIL and LOCF. However, when assuming dropout due to deteriorating health conditions in 2Stage ($k$= 1.2) and 2 stage ($k$= 1.1), the estimated HRs indicated that higher Composite- 5 predicted a reduced hazard for mortality. Notably, the lowest HR of 0.8 (95% CI 0.76 $-$ 0.85) was observed in 2Stage ($k$= 1.2). These results resembled the findings obtained from CC-exam5, CC-exam6 and CC-exam7, which had relatively low dropout rates. On the contrary, 2Stage ($k$= 0.9) and 2Stage ($k$= 0.8), which assumed dropout due to improved health, produced a higher estimated mortality hazard for each incremental increase in Composite- 5, with the largest estimated HR reaching 1.26 (95% CI 1.20 $-$ 1.33). Sensitivity analyses that treated the participants who missed the final exam as MAR produced results similar to those of our main analyses. (Supplementary Material 1: Figure S9 and Figure S10).

## Discussion

We evaluated the two-stage MI framework to impute missing values due to two mechanisms, MAR and MNAR. Within this framework, we imputed missing values in the continuous and binary components to construct a multi-component composite variable in a longitudinal setting. Additionally, we compared three FCS methods. We assessed the mean and slope across time points for this composite variable. We also evaluated HR for mortality for the longitudinal composite variable after imputation under various ignorability assumptions. Simulation studies indicated that two-stage MI under appropriately specified ignorability assumptions produced the smallest bias and optimal coverage. Two-stage MI assuming MAR yielded biased estimates fell between those of appropriately specified ignorability assumptions and the misspecified ones. The FCS methods incorporating longitudinal information within the two-stage MI framework yielded the best results in most scenarios, outperforming CC analysis and LOCF. AFCS and LFCS performed similarly in our simulation. However, the more parsimonious LFCS is preferable due to its lower computational burden and straightforward interpretation.

The application in the FHS Offspring cohort further suggested that the three parameters, the mean, slope across time points, and mortality HR for Composite- 5, were influenced by the choice of ignorability assumptions. Under the assumption of dropout due to deteriorating health, Composite- 5 imputed within two-stage MI showed a declining trend over time and a protective effect against mortality. In contrast, assuming MAR for dropout resulted in an increasing trend in Composite- 5 over time and showed no significant association with mortality. The three FCS methods differed in bias and coverage probability in simulation, but they performed similarly when applied to the FHS data. This discrepancy is likely due to the differences in missing data patterns between the simulated and real datasets. In simulation, non-attendance (except for death) was generated for each exam separately, resulting in fewer dropout patterns than the real data. The real data had more dropout patterns, leading to reduced longitudinal information that is needed in AFCS and LFCS. As a result, the three FCS methods tended to perform similarly in the real data.

Our findings were consistent with prior studies that used similar approaches. Using traditional MI, Kane (2017) imputed missing values in the components of a longitudinal composite variable and compared several FCS methods [14]. The FCS models that considered longitudinal information produced unbiased estimates under MAR and MCAR but failed to account for MNAR. Boyko (2013) proposed a three-stage MI framework to impute missing values due to MCAR, MAR and MNAR in three different stages [16]. They evaluated various ignorability assumptions and demonstrated that misspecification of these assumptions led to substantial bias [16]. In contrast to our study, the three-stage method solely focused on continuous variables and did not compare imputation methods within the framework.

The constant multiplier *k* used in two-stage MI has been shown to be both reasonable and effective. First, this method assumes a scale bias between the true distribution of underlying missing values and the distribution of observed values. We observed such bias in the FHS data, as cardiovascular health differed between participants who dropped out and those who remained. Second, the method of a constant multiplier *k* may work better for a composite variable compared to the univariate case. The binary indicators that are converted from individual components are less sensitive to the specific values of the multiplier *k* than original components. As long as the direction (e.g., *k* > 1 versus *k* < 1) of MNAR imputation is correct, the imputed components produce reasonable binary indicators, leading to a reasonable Composite- 5 score.

We performed two-stage MI on the individual components before transforming them to binary indicators and Composite- 5 due to the following reasons. First, imputing binary indicator variables can lead to potentially misleading results. For instance, DBP values of 81 and 120 both yield a binary indicator of 0. A binary indicator treats these two distinct values the same during imputation, which could result in inaccurate results. Second, previous studies have discussed the comparison between item-level (e.g., components) and scale-level (e.g., composite score) imputation and suggested that item-level imputation may be more appropriate [22–24]. Unlike scale-level imputation, item-level imputation typically incorporates other observed item-level responses, which tend to make the imputation more accurate.

We evaluated three FCS methods in this study, as different methods may be better suited to different scenarios. When both within-component correlations across exams and between-component correlations are high, AFCS is more desirable since it incorporates both longitudinal and cross-sectional information. When within-component correlations across exams are high but between-component correlations are low, AFCS and LFCS perform similarly. However, AFCS is more computationally intensive, especially as the number of time points increases. In such cases, the more parsimonious LFCS model is preferable for its low computational burden and easier interpretation. Similarly, when between-component correlations are high but within-component correlations across exams are low, XFCS may be more appropriate.

To address MNAR data, commonly used methods include likelihood-based approaches that specify the joint distribution of the data and the missing data mechanisms, such as selection models and pattern mixture models [1]. Specific MI methods have also been proposed to address MNAR by investigating departures from the assumption of ignorability [25–27], many of which are based on the pattern-mixture model framework. Our two-stage MI is a natural extension of the pattern mixture model by allowing different ignorability assumptions for different missing patterns. Unlike previous likelihood-based methods, two-stage MI is more flexible, allowing for standard complete-data statistical methods. Compared to other MI methods for MNAR data, two-stage MI explicitly distinguishes between missingness due to MAR and MNAR, and accounts for the uncertainty arising from the imputation of both mechanisms. Additionally, it accommodates different data types in a composite variable.

Our study has limitations. As a simplified version of Life’s Simple 7 [28], we used the self-defined Composite- 5 in which physical activity and diet were not included due to data unavailability across exams. Therefore, Composite- 5 may not provide a comprehensive assessment of the overall cardiovascular health as Life's Simple 7. Second, our sensitivity analyses with a range of ignorability assumptions resulted in a range of answers rather than a single inference, which may add complexity to interpretation [29]. However, sensitivity analysis is an essential part of our model, which enhances the robustness and comprehensiveness of our conclusions by exploring the impact of varying assumptions and evaluating whether the results are driven by model assumptions.

The following next steps may be considered. First, Life’s Simple 7 can be evaluated within the two-stage framework as more data, including categorical variables, are collected in the FHS. Second, when handling missing values in repeated measures at irregular time intervals, extensions of standard FCS should be considered, such as the FCS methods based on linear mixed models [20]. Third, the current framework may be extended to integrate model uncertainty regarding ignorability assumptions using a three-stage MI framework [16], where model uncertainty can be modeled using a multiplier $k$ drawn from a distribution in one of the three stages. The incorporation of the uncertainty regarding ignorability assumptions allows researchers to integrate a range of multiplier $k$ into one inference and reduce the strength of the assumption being made [8, 9]. Fourth, instead of using a multiplier $k$, imputation methods based on selection models or pattern mixture models may also be employed to handle missing data due to MNAR within the second step of Stage 1 in the two-stage MI framework. Lastly, the current study can be extended to evaluate rates of missing information. Previous studies discussed rates of missing information for two-stage MI in the univariate case [3, 7], but not with a composite score.

## Conclusions

In the context of a longitudinal composite variable with missing values due to various missing mechanisms within the two-stage MI framework, we demonstrated that the FCS methods, integrating longitudinal information while assuming appropriate ignorability assumptions, exhibited the most optimal performance. Our study underscored the importance of selecting appropriate imputation methods and ignorability assumptions within the two-stage MI framework. Our study featured an innovative utilization of two-stage MI, a method with immense potential yet limited application and extension within the field.

## Supplementary Information

Supplementary Material 1

## Data Availability

The individual components for constructing the Composite- 5 index can be requested at https://www.ncbi.nlm.nih.gov/projects/gap/cgi-bin/document.cgi?study_id=phs000007.v33.p14&phv=523294&phd=4398&pha=4313&pht=12882&phvf=&phdf=&phaf=&phtf=&dssp=1&consent=&temp=1. Simulated data and code during the current study are available from the following GitHub page https://github.com/xzwang19/2StageComposite.

## Abbreviations

MCAR: Missing completely at random
MAR: Missing at random
MNAR: Missing not at random
MI: Multiple imputation
FCS: Fully conditional specification
AFCS: All fully conditional specification
LFCS: Longitudinal fully conditional specification
XFCS: Cross-sectional fully conditional specification
SBP: Systolic blood pressure
DBP: Diastolic blood pressure
TC: Total cholesterol
ANOVA: Analysis of variance
FHS: Framingham Heart Study
LME: Linear mixed-effects model
CC: Complete case
HR: Hazard ratio
