# Supplementary material for: Two-stage multiple imputation with a longitudinal composite variable
Source: BMC Med Res Methodol. 2025 May 6;25:124. doi: 10.1186/s12874-025-02555-9 (PMC12054270; doi:10.1186/s12874-025-02555-9)
Supplement: Supplementary file 1 — Supplementary Material 1 [file 12874_2025_2555_MOESM1_ESM.docx]

**Two-stage multiple imputation with a longitudinal composite variable**

**(Additional File 1)**

Xuzhi Wang^1^, Martin G. Larson^1^, Chunyu Liu^1^

1. Department of Biostatistics, Boston University School of Public Health, Boston MA 02118

Corresponding author: Xuzhi Wang ([xwang19@bu.edu](mailto:xwang19@bu.edu))

**Methods**

**Method S1. Steps for generating event times.**

We simulated time to death for each 5-year time interval using piecewise exponential models with parameters that mimicked the FHS Offspring cohort data with the following steps:

**Step 1**. Let $t_{ij}$ denote the survival times since each time point $j$ for participant $i$ ($j=1,2,3,4,5, i=1,2,\ldots,3700).$ $t_{ij}$ was generated from an exponential distribution $Exp(\lambda_{ij})$ with $\lambda_{ij}=\lambda_{j}\exp\left( X_{ij}\beta\right)=\lambda_{j}exp(\beta_{1}C_{ij}+\beta_{2}{sex}_{i}+\beta_{3}{age}_{i})$, where $C_{ij}$ denoted the score of the composite variable at time point $j$ for participant $i$, and $sex_{i}$ and $age_{i}$ were sex and baseline age for participant $i$. The parameter values were specified based on the real data with $\left( \beta_{1}, \beta_{2},\beta_{3} \right)=(-0.22, 0.11, -0.54)$, indicating that a higher risk of death was associated with a smaller value of composite variable. The values of $\lambda_{j}$ were configured such that the proportion of mortality were around 2% between baseline and year 5, 3% between year 5 and year 10, 5% between year 10 and year 15, 10% between year 15 and year 20, and 20% between year 20 and year 25.

**Step 2**. We imposed the following constraints to each five-year time interval: $t_{j}=min\{t_{j}, 5\}$, and $t_{j+1}=0$ if $t_{j}<5$. The overall observed survival time for participant $i$ was $T_{i}=t_{1}+t_{2}+t_{3}+t_{4}+t_{5}$ so that the longest follow-up time was 25 years (5 years after the last exam). $D_{i}=I\{T_{i}\leq25\}$ denoted death indicator for participant $i$.

**Step 3**. For each participant $i$, the longitudinal individual components that occurred after time $T_{i}$ were dropped.

**Tables**

**Table S1. Components in Composite-5.**

| **Panel A** | | |
| --- | --- | --- |
| **Components** | **Not Ideal** | **Ideal** |
| **Smoking status** | Current Smoker or quit smoking less the 12 months ago (smoking status = 1) | Never or non-smoker for over a year (smoking status = 0) |
| **BMI** | BMI ≥ 25 | BMI less than 25 |
| **Blood pressure** | SBP ≥ 120 or DBP ≥ 80 mmHg | SBP < 120 and DBP < 80 mmHg |
| **Total cholesterol** | Total cholesterol ≥200 mg/dL | Total cholesterol less than 200 mg/dL |
| **Blood glucose** | Fasting plasma glucose ≥ 100 mg/dL | Fasting plasma glucose less than 100 mg/dL |
| **Panel B** | | |
| **Indicator score for each component** | 0 | 1 |
| **Total score range for Composite-5** | [0,5] | |

**Panel A** shows the criteria used to classify “ideal” and “not ideal” for each component. **Panel B** shows the indicator score for each component based on these criteria, as well as the total score range for Composite-5. Composite-5 was constructed by summing up the indicator scores (i.e., 0 or 1) for each of the five components. BMI, body mass index; SBP, systolic blood pressure; DBP, diastolic blood pressure

**Table S2. Mean and standard deviation of the simulated data.**

| **Variable** | **Exam** | **Mean** | **SD** |
| --- | --- | --- | --- |
| Age | 1 | 55 | 10 |
| Sex |  | .5 | .5 |
| Smoking | 1 | .12 | .3 |
|  | 2 | .14 | .3 |
|  | 3 | .16 | .3 |
|  | 4 | .18 | .3 |
|  | 5 | .2 | .3 |
| BMI | 1 | 27 | 5 |
|  | 2 | 28 | 5 |
|  | 3 | 29 | 5 |
|  | 4 | 30 | 5 |
|  | 5 | 31 | 5 |
| SBP | 1 | 130 | 19 |
|  | 2 | 130 | 19 |
|  | 3 | 130 | 19 |
|  | 4 | 130 | 19 |
|  | 5 | 130 | 19 |
| DBP | 1 | 74 | 10 |
|  | 2 | 75 | 10 |
|  | 3 | 76 | 10 |
|  | 4 | 76 | 10 |
|  | 5 | 76 | 10 |
| TC | 1 | 200 | 37 |
|  | 2 | 202 | 37 |
|  | 3 | 204 | 37 |
|  | 4 | 206 | 37 |
|  | 5 | 208 | 37 |
| BG | 1 | 100 | 29 |
|  | 2 | 102 | 29 |
|  | 3 | 104 | 29 |
|  | 4 | 106 | 29 |
|  | 5 | 108 | 29 |

BMI, body mass index; SBP, systolic blood pressure; DBP, diastolic blood pressure; TC, total cholesterol; BG, fasting blood glucose.

**Table S3. Correlation structure of the simulated data.**

| Variable and exam | | Smoking | | | | | BMI | | | | | SBP | | | | | DBP | | | | | TC | | | | | BG | | | | |
| --- | --- | --- | --- | --- | --- | --- | --- | --- | --- | --- | --- | --- | --- | --- | --- | --- | --- | --- | --- | --- | --- | --- | --- | --- | --- | --- | --- | --- | --- | --- | --- |
|  |  | 1 | 2 | 3 | 4 | 5 | 1 | 2 | 3 | 4 | 5 | 1 | 2 | 3 | 4 | 5 | 1 | 2 | 3 | 4 | 5 | 1 | 2 | 3 | 4 | 5 | 1 | 2 | 3 | 4 | 5 |
| Age | 1 | -.15 | -.15 | -.15 | -.15 | -.15 | .05 | .04 | .03 | .02 | .01 | .3 | | | | | .1 | .1 | -.1 | -.2 | -.3 | .25 | .2 | .15 | .1 | .05 | .1 | | | | |
| Sex |  | 0 | | | | | -.2 | -.15 | -.1 | -.1 | -.1 | -.2 | -.15 | -.1 | -.5 | 0 | -.25 | -.2 | -.15 | -.1 | -.05 | .05 | .1 | .15 | .2 | .25 | -.2 | | | | |
| Smoking | 1 | 1 | .8 | .7 | .6 | .5 | -.05 | | | | | -.1 | | | | | -.05 | | | | | 0 | | | | | .05 | | | | |
|  | 2 |  | 1 | .8 | .7 | .6 |  |  |  |  |  |  |  |  |  |  |  |  |  |  |  |  |  |  |  |  |  |  |  |  |  |
|  | 3 |  |  | 1 | .8 | .7 |  |  |  |  |  |  |  |  |  |  |  |  |  |  |  |  |  |  |  |  |  |  |  |  |  |
|  | 4 |  |  |  | 1 | .8 |  |  |  |  |  |  |  |  |  |  |  |  |  |  |  |  |  |  |  |  |  |  |  |  |  |
|  | 5 |  |  |  |  | 1 |  |  |  |  |  |  |  |  |  |  |  |  |  |  |  |  |  |  |  |  |  |  |  |  |  |
| BMI | 1 |  |  |  |  |  | 1 | .95 | .9 | .85 | .8 | .2 | | | | | .15 | | | | | -.05 | | | | | .25 | | | | |
|  | 2 |  |  |  |  |  |  | 1 | .95 | .9 | .85 |  |  |  |  |  |  |  |  |  |  |  |  |  |  |  |  |  |  |  |  |
|  | 3 |  |  |  |  |  |  |  | 1 | .95 | .9 |  |  |  |  |  |  |  |  |  |  |  |  |  |  |  |  |  |  |  |  |
|  | 4 |  |  |  |  |  |  |  |  | 1 | .95 |  |  |  |  |  |  |  |  |  |  |  |  |  |  |  |  |  |  |  |  |
|  | 5 |  |  |  |  |  |  |  |  |  | 1 |  |  |  |  |  |  |  |  |  |  |  |  |  |  |  |  |  |  |  |  |
| SBP | 1 |  |  |  |  |  |  |  |  |  |  | 1 | .65 | .55 | .45 | .35 | .5 | .4 | .3 | .2 | .1 | .05 | | | | | .15 | | | | |
|  | 2 |  |  |  |  |  |  |  |  |  |  |  | 1 | .65 | .55 | .45 | .4 | .5 | .4 | .3 | .2 |  |  |  |  |  |  |  |  |  |  |
|  | 3 |  |  |  |  |  |  |  |  |  |  |  |  | 1 | .65 | .55 | .3 | .4 | .5 | .4 | .3 |  |  |  |  |  |  |  |  |  |  |
|  | 4 |  |  |  |  |  |  |  |  |  |  |  |  |  | 1 | .65 | .2 | .3 | .4 | .5 | .4 |  |  |  |  |  |  |  |  |  |  |
|  | 5 |  |  |  |  |  |  |  |  |  |  |  |  |  |  | 1 | .1 | .2 | .3 | .4 | .5 |  |  |  |  |  |  |  |  |  |  |
| DBP | 1 |  |  |  |  |  |  |  |  |  |  |  |  |  |  |  | 1 | .55 | .45 | .35 | .25 | .05 | | | | | .05 | | | | |
|  | 2 |  |  |  |  |  |  |  |  |  |  |  |  |  |  |  |  | 1 | .55 | .45 | .35 |  |  |  |  |  |  |  |  |  |  |
|  | 3 |  |  |  |  |  |  |  |  |  |  |  |  |  |  |  |  |  | 1 | .55 | .45 |  |  |  |  |  |  |  |  |  |  |
|  | 4 |  |  |  |  |  |  |  |  |  |  |  |  |  |  |  |  |  |  | 1 | .55 |  |  |  |  |  |  |  |  |  |  |
|  | 5 |  |  |  |  |  |  |  |  |  |  |  |  |  |  |  |  |  |  |  | 1 |  |  |  |  |  |  |  |  |  |  |
| TC | 1 |  |  |  |  |  |  |  |  |  |  |  |  |  |  |  |  |  |  |  |  | 1 | .6 | .45 | .3 | .15 | -.05 | | | | |
|  | 2 |  |  |  |  |  |  |  |  |  |  |  |  |  |  |  |  |  |  |  |  |  | 1 | .6 | .45 | .3 |  |  |  |  |  |
|  | 3 |  |  |  |  |  |  |  |  |  |  |  |  |  |  |  |  |  |  |  |  |  |  | 1 | .6 | .45 |  |  |  |  |  |
|  | 4 |  |  |  |  |  |  |  |  |  |  |  |  |  |  |  |  |  |  |  |  |  |  |  | 1 | .6 |  |  |  |  |  |
|  | 5 |  |  |  |  |  |  |  |  |  |  |  |  |  |  |  |  |  |  |  |  |  |  |  |  | 1 |  |  |  |  |  |
| BG | 1 |  |  |  |  |  |  |  |  |  |  |  |  |  |  |  |  |  |  |  |  |  |  |  |  |  | 1 | .7 | .6 | .5 | .4 |
|  | 2 |  |  |  |  |  |  |  |  |  |  |  |  |  |  |  |  |  |  |  |  |  |  |  |  |  |  | 1 | .7 | .6 | .5 |
|  | 3 |  |  |  |  |  |  |  |  |  |  |  |  |  |  |  |  |  |  |  |  |  |  |  |  |  |  |  | 1 | .7 | .6 |
|  | 4 |  |  |  |  |  |  |  |  |  |  |  |  |  |  |  |  |  |  |  |  |  |  |  |  |  |  |  |  | 1 | .7 |
|  | 5 |  |  |  |  |  |  |  |  |  |  |  |  |  |  |  |  |  |  |  |  |  |  |  |  |  |  |  |  |  | 1 |

BMI, body mass index; SBP, systolic blood pressure; DBP, diastolic blood pressure; TC, total cholesterol; BG, fasting blood glucose.

**Table S4. Coefficients in logistic regressions for data missing due to MAR.**

| **Panel 1** | | | | |
| --- | --- | --- | --- | --- |
|  | $\boldsymbol{\beta}_{\boldsymbol{0}}$ | $\boldsymbol{\beta}_{\boldsymbol{1}}$ | $\boldsymbol{\beta}_{\boldsymbol{2}}$ | $\boldsymbol{\beta}_{\boldsymbol{3}}$ |
| **Time 2** | -3.2435 | 0.0029 | 0.014 | -0.0854 |
| **Time 3** | -4.1538 | 0.0060 | 0.0289 | -0.2885 |
| **Time 4** | -5.3353 | 0.0081 | 0.06 | -0.322 |
| **Time 5** | -5.9439 | 0.0097 | 0.0795 | -0.2528 |
| **Panel 2** | | | | |
|  | $\boldsymbol{\gamma}_{\boldsymbol{0}}$ | $\boldsymbol{\gamma}_{\boldsymbol{1}}$ | $\boldsymbol{\gamma}_{\boldsymbol{2}}$ | $\boldsymbol{\gamma}_{\boldsymbol{3}}$ |
| **Time 2** | -2.6521 | -0.0815 | 0.0137 | -0.0644 |
| **Time 3** | -3.0566 | -0.1368 | 0.0287 | -0.2563 |
| **Time 4** | -3.8709 | -0.1973 | 0.0608 | -0.2854 |
| **Time 5** | -4.1916 | -0.2292 | 0.0802 | -0.2029 |

Panel A shows the logistic model for the MAR mechanism of SBP missingness: $log\left( \frac{Pr\left( missing \right)}{1-Pr\left( missing \right)} \right)=\beta_{0}+\beta_{1}SBP_{1}+\beta_{2}AGE_{1}+\beta_{3}SEX$, where $SBP_{1}$ is SBP at baseline, $AGE_{1}$ is age at baseline, and $SEX$ is a binary indicator for sex. Notably, when SBP is missing at a particular time point, we assume DBP is also missing at that time point.

Panel B shows the logistic model for the MAR missingness of non-attendance: $log\left( \frac{Pr\left( missing \right)}{1-Pr\left( missing \right)} \right)=\gamma_{0}+\gamma_{1}COMPOSITE_{1}+\gamma_{2}AGE_{1}+\gamma_{3}SEX$, where $COMPOSITE_{1}$ is the value of the composite variable at baseline, $AGE_{1}$ is age at baseline, and $SEX$ is a binary indicator for sex.

**Figures**

**
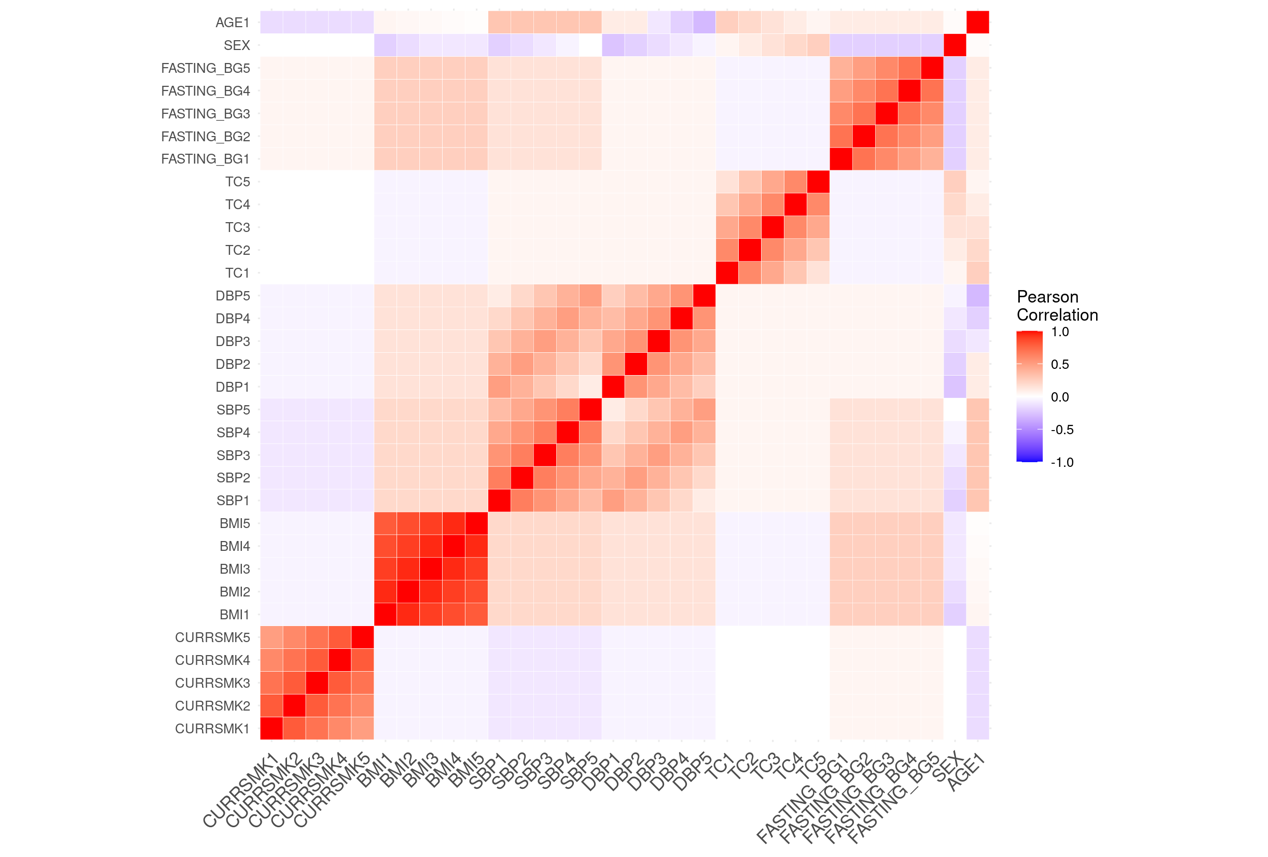
**

**Figure S1. Correlation heatmap of individual components in the composite variable.** BMI, body mass index; SBP, systolic blood pressure; DBP, diastolic blood pressure; TC, total cholesterol; BG, fasting blood glucose.

**
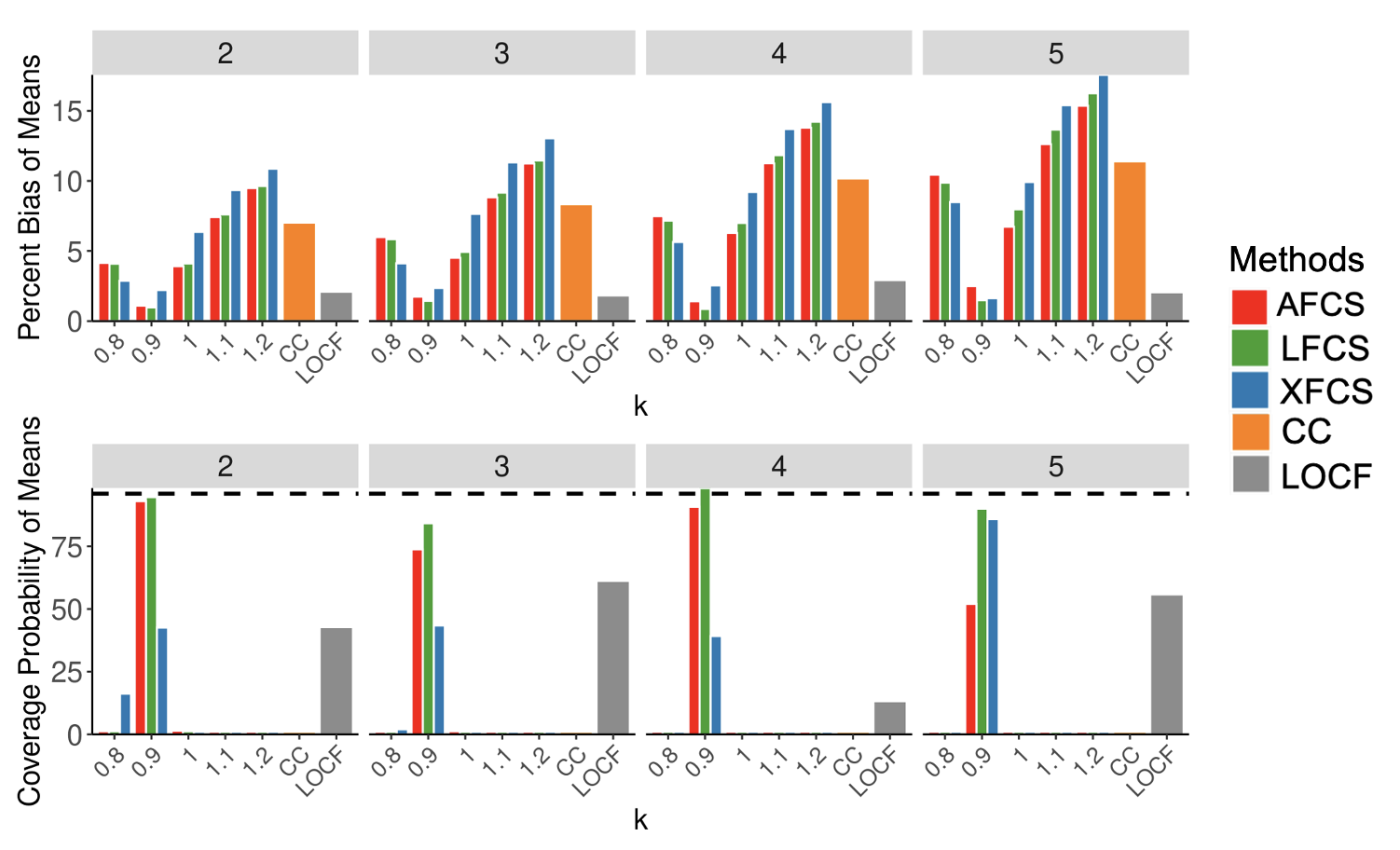
**

**Figure S2. Simulation results for the mean of the composite variable in Scenario 2.** The x-axis in each panel denotes various ignorability assumptions in two-stage MI. The top panel displays the percent bias of means (%), while the bottom panel illustrates the coverage probability of means (%). Four subpanels in each of the two panels represent four time points. The dashed line in the lower panel is the 95% coverage probability. $k$=0.8 and 0.9 represent the appropriately specified MNAR assumption, $k$ =1 represents the MAR assumption, $k$ =1.1 and 1.2 represent the misspecified MNAR assumption. MI, multiple imputation; MAR, missing at random; MNAR, missing not at random; AFCS, all fully conditional specification; CC, complete case analysis; LFCS, longitudinal fully conditional specification; XFCS, cross-sectional fully conditional specification; LOCF, last observation carried forward.

**
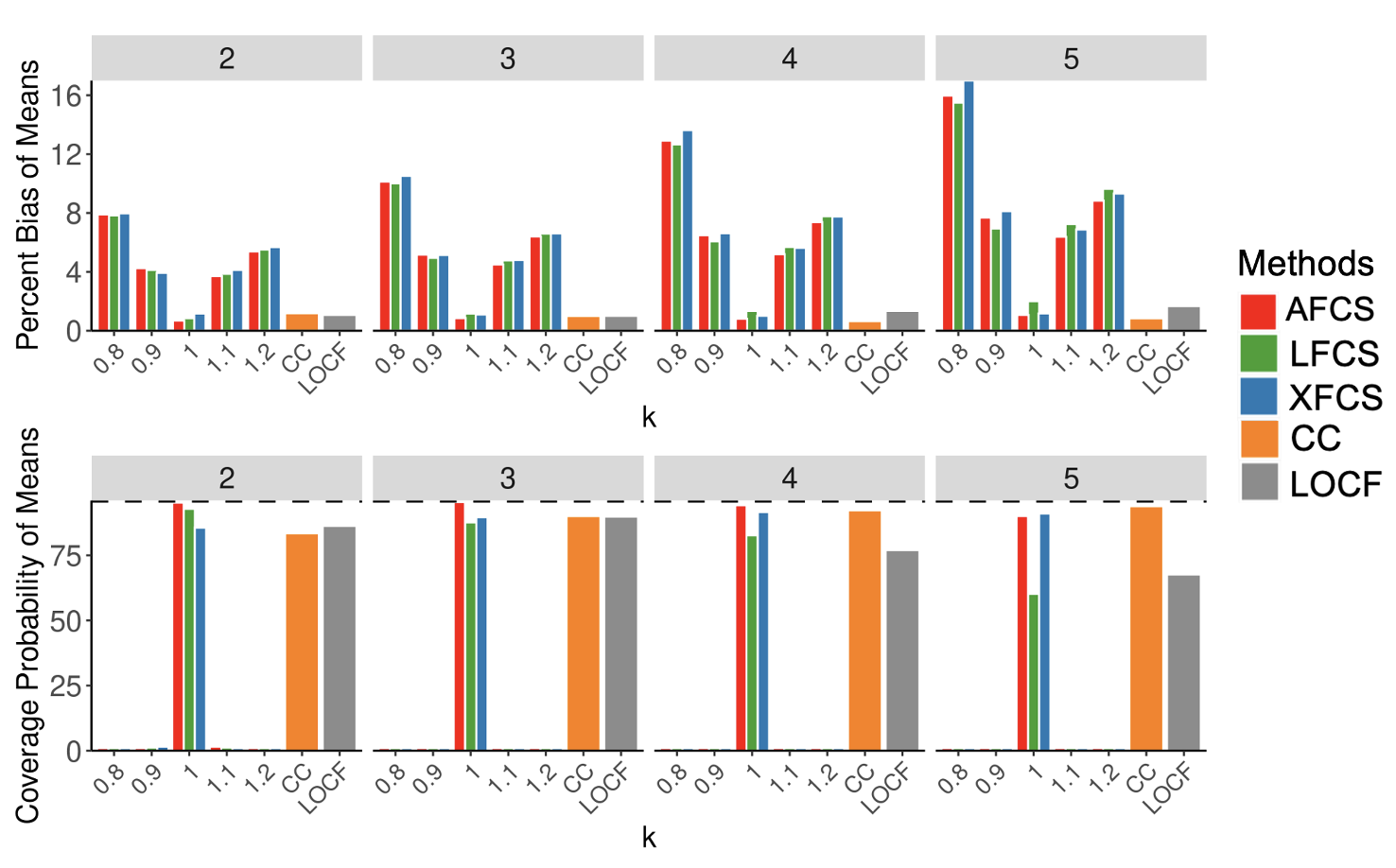
**

**Figure S3. Simulation results for the mean of the composite variable in Scenario 3.** The x-axis in each panel denotes various ignorability assumptions in two-stage MI. The top panel displays the percent bias of means (%), while the bottom panel illustrates the coverage probability of means (%). Four subpanels in each of the two panels represent four time points. The dashed line in the lower panel is the 95% coverage probability. $k$=0.8, 0.9, 1.1, and 1.2 represent the misspecified MNAR assumption, $k$ =1 represents the MAR assumption. MI, multiple imputation; MAR, missing at random; MNAR, missing not at random; AFCS, all fully conditional specification; CC, complete case analysis; LFCS, longitudinal fully conditional specification; XFCS, cross-sectional fully conditional specification; LOCF, last observation carried forward.


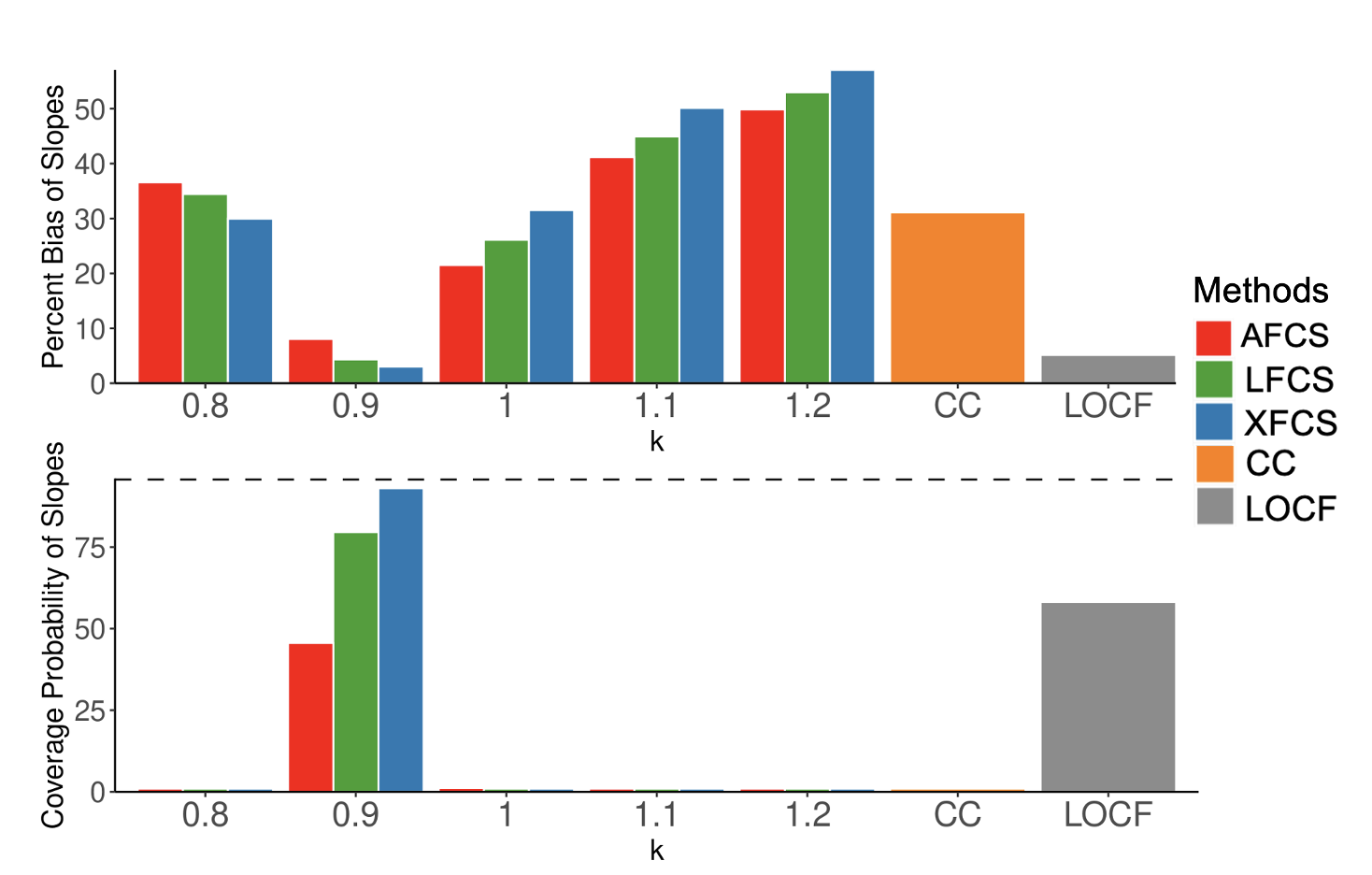


**Figure S4. Simulation results for the slope of the composite variable in Scenario 2.** The x-axis in each panel denotes various ignorability assumptions in two-stage MI. The top panel displays the percent bias of slope (%), while the bottom panel illustrates the coverage probability of slope (%). The dashed line in the lower panel is the 95% coverage probability. $k$=0.8 and 0.9 represent the appropriately specified MNAR assumption, $k$ =1 represents the MAR assumption, $k$ =1.1 and 1.2 represent the misspecified MNAR assumption. MI, multiple imputation; MAR, missing at random; MNAR, missing not at random; AFCS, all fully conditional specification; CC, complete case analysis; LFCS, longitudinal fully conditional specification; XFCS, cross-sectional fully conditional specification; LOCF, last observation carried forward.


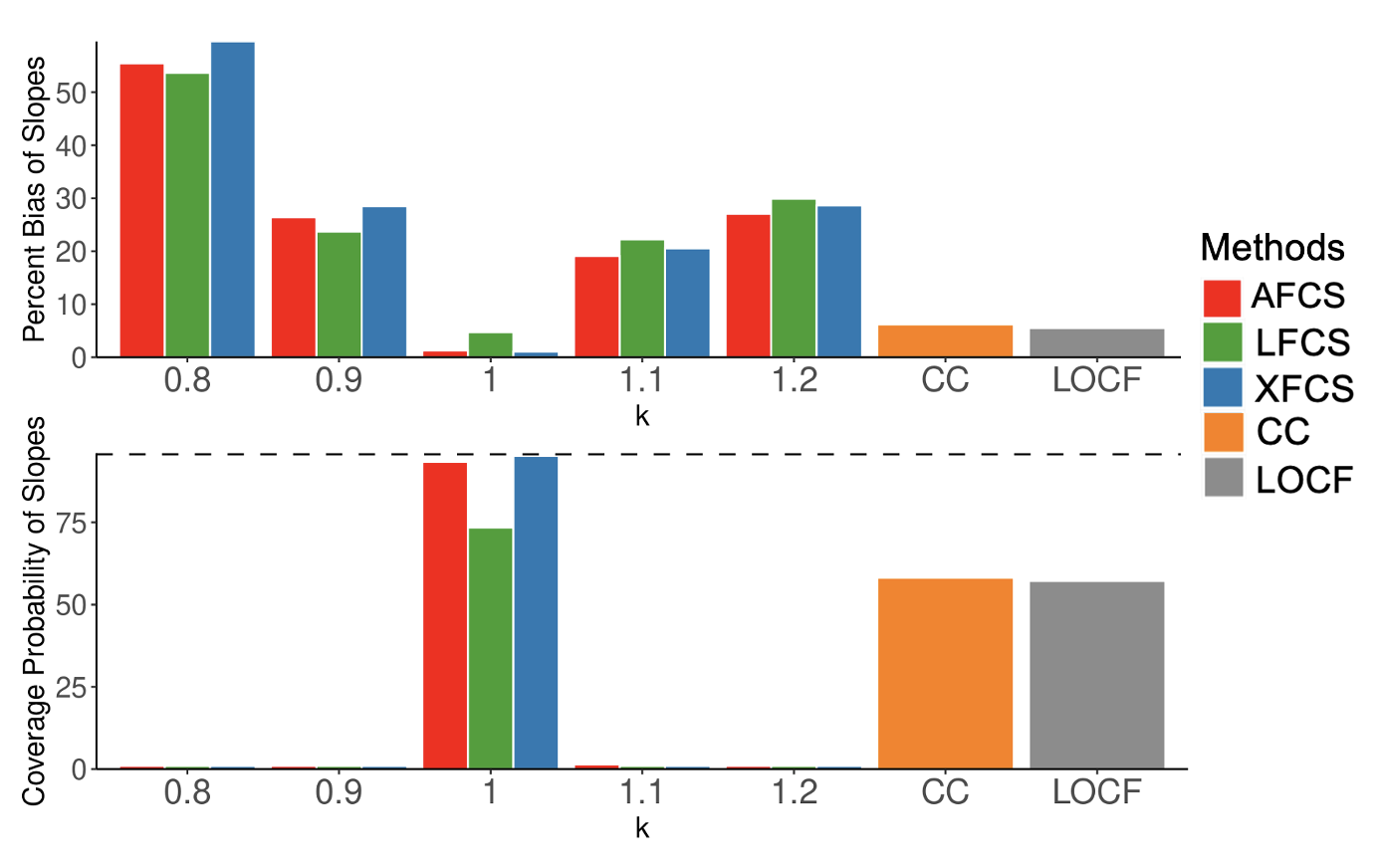


**Figure S5. Simulation results for the slope of the composite variable in Scenario 3.** The x-axis in each panel denotes various ignorability assumptions in two-stage MI. The top panel displays the percent bias of slope (%), while the bottom panel illustrates the coverage probability of slope (%). The dashed line in the lower panel is the 95% coverage probability. $k$ =1 represents the MAR assumption, $k$ =0.8, 0.9, 1.1 and 1.2 represent the misspecified MNAR assumption. MI, multiple imputation; MAR, missing at random; MNAR, missing not at random; AFCS, all fully conditional specification; CC, complete case analysis; LFCS, longitudinal fully conditional specification; XFCS, cross-sectional fully conditional specification; LOCF, last observation carried forward.


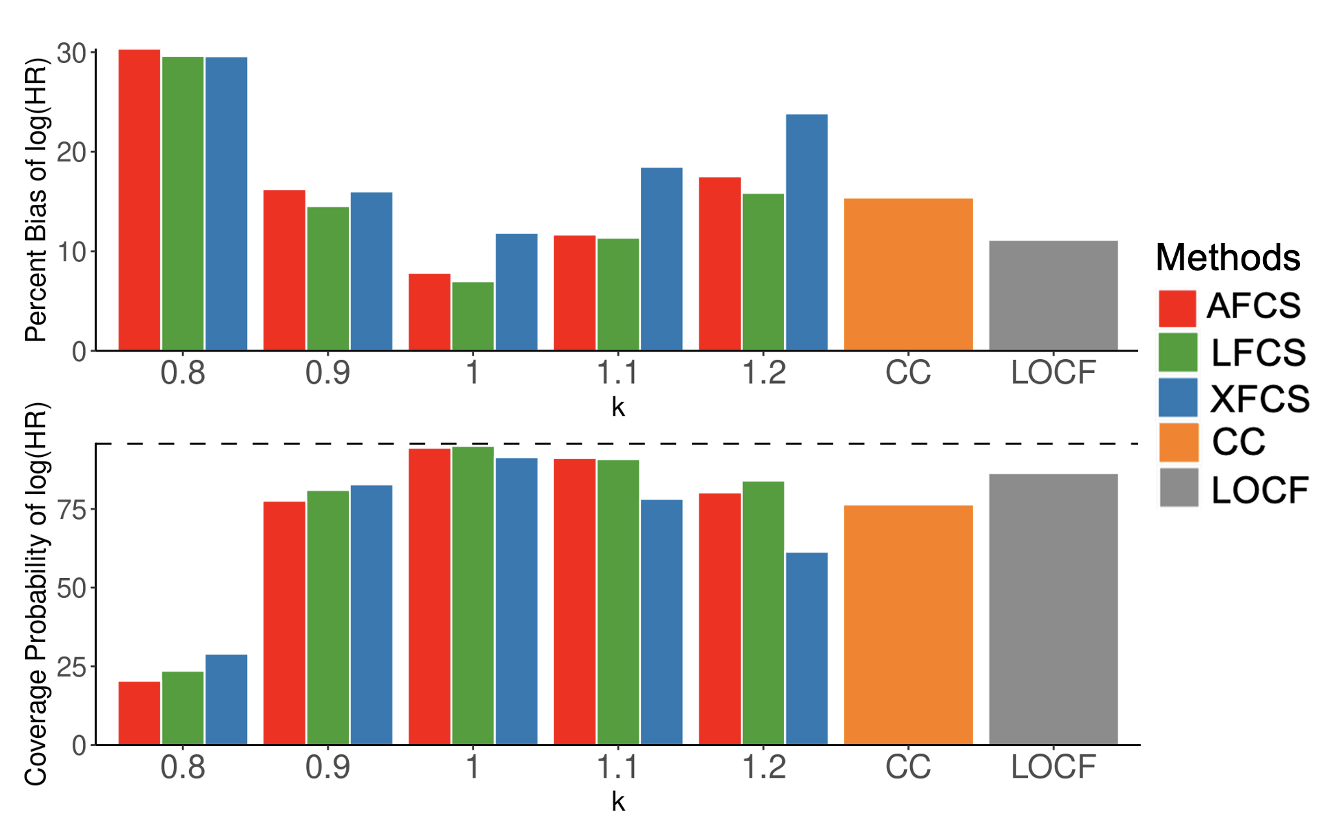


**Figure S6. Simulation results for log HR of the composite variable in Scenario 2.** The x-axis in each panel denotes various ignorability assumptions in two-stage MI. The top panel displays the percent bias of log HR (%), while the bottom panel illustrates the coverage probability of log HR (%). The dashed line in the lower panel is the 95% coverage probability. $k$ =1 represents the MAR assumption, $k$ =0.8, 0.9, 1.1 and 1.2 represent the misspecified MNAR assumption. MI, multiple imputation; MAR, missing at random; MNAR, missing not at random; AFCS, all fully conditional specification; CC, complete case analysis; LFCS, longitudinal fully conditional specification; XFCS, cross-sectional fully conditional specification; LOCF, last observation carried forward.


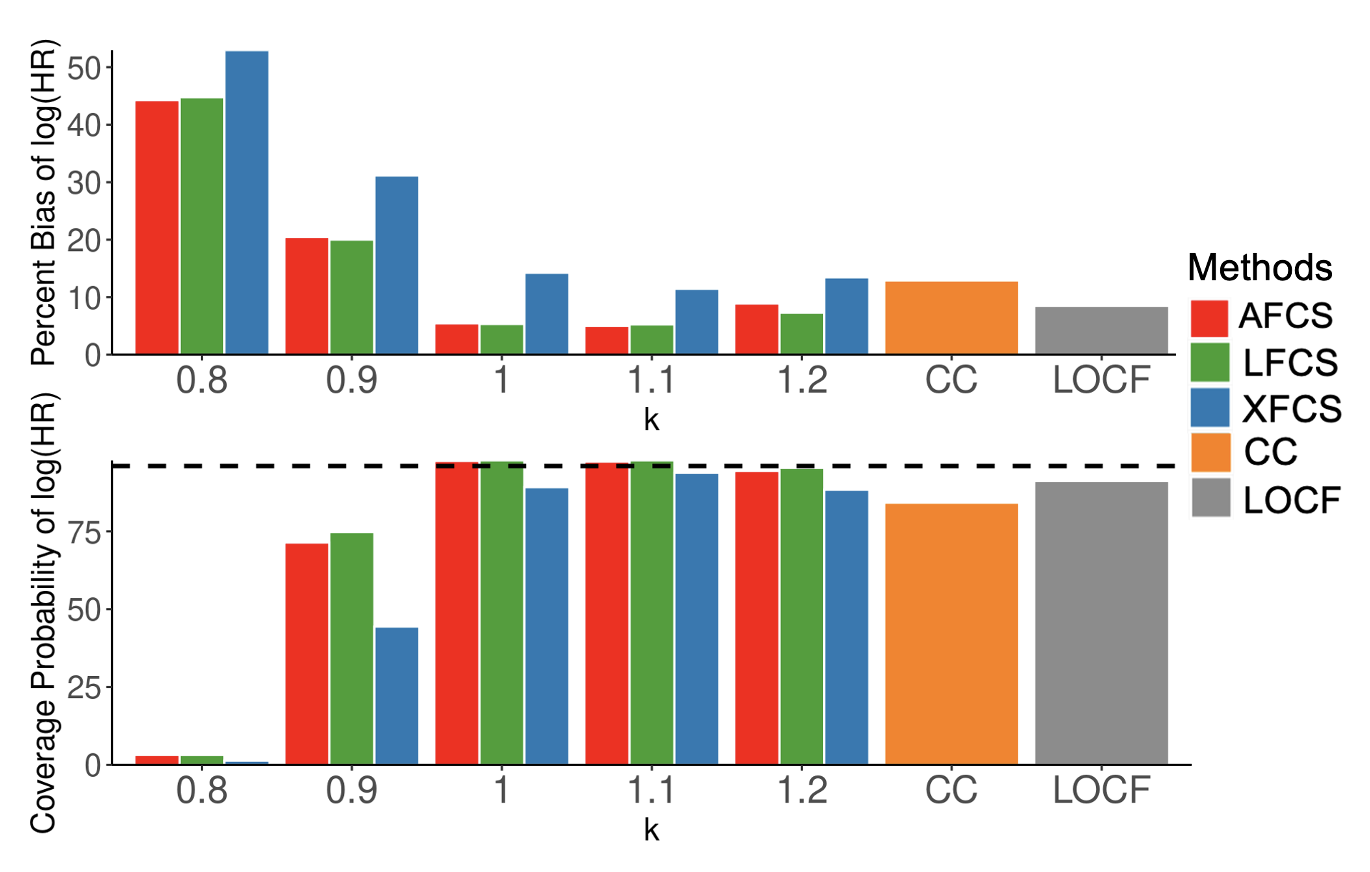


**Figure S7. Simulation results for log HR of the composite variable in Scenario 3.** The x-axis in each panel denotes various ignorability assumptions in two-stage MI. The top panel displays the percent bias of log HR (%), while the bottom panel illustrates the coverage probability of log HR (%). The dashed line in the lower panel is the 95% coverage probability. $k$ =1 represents the MAR assumption, $k$ =0.8, 0.9, 1.1 and 1.2 represent the misspecified MNAR assumption. MI, multiple imputation; MAR, missing at random; MNAR, missing not at random; AFCS, all fully conditional specification; CC, complete case analysis; LFCS, longitudinal fully conditional specification; XFCS, cross-sectional fully conditional specification; LOCF, last observation carried forward.


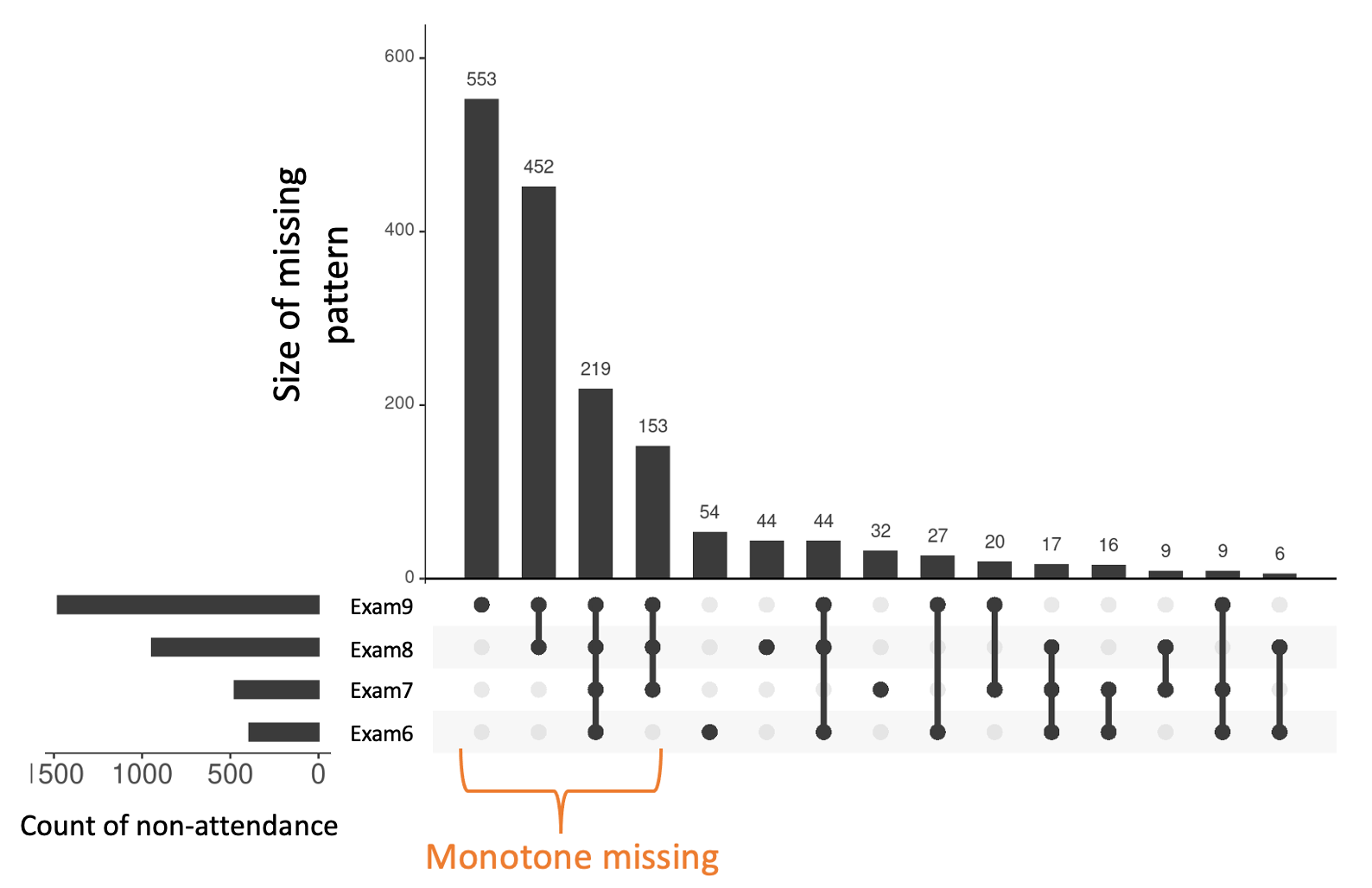


**Figure S8. Missing patterns in the FHS Offspring cohort**. Each black dot represents non-attendance at that exam. The upper panel shows the frequencies of each missing pattern, and the lower panel displays the missing patterns from exam 5 to exam 9. The panel on the bottom left displays the count of non-attendance at each exam.

**
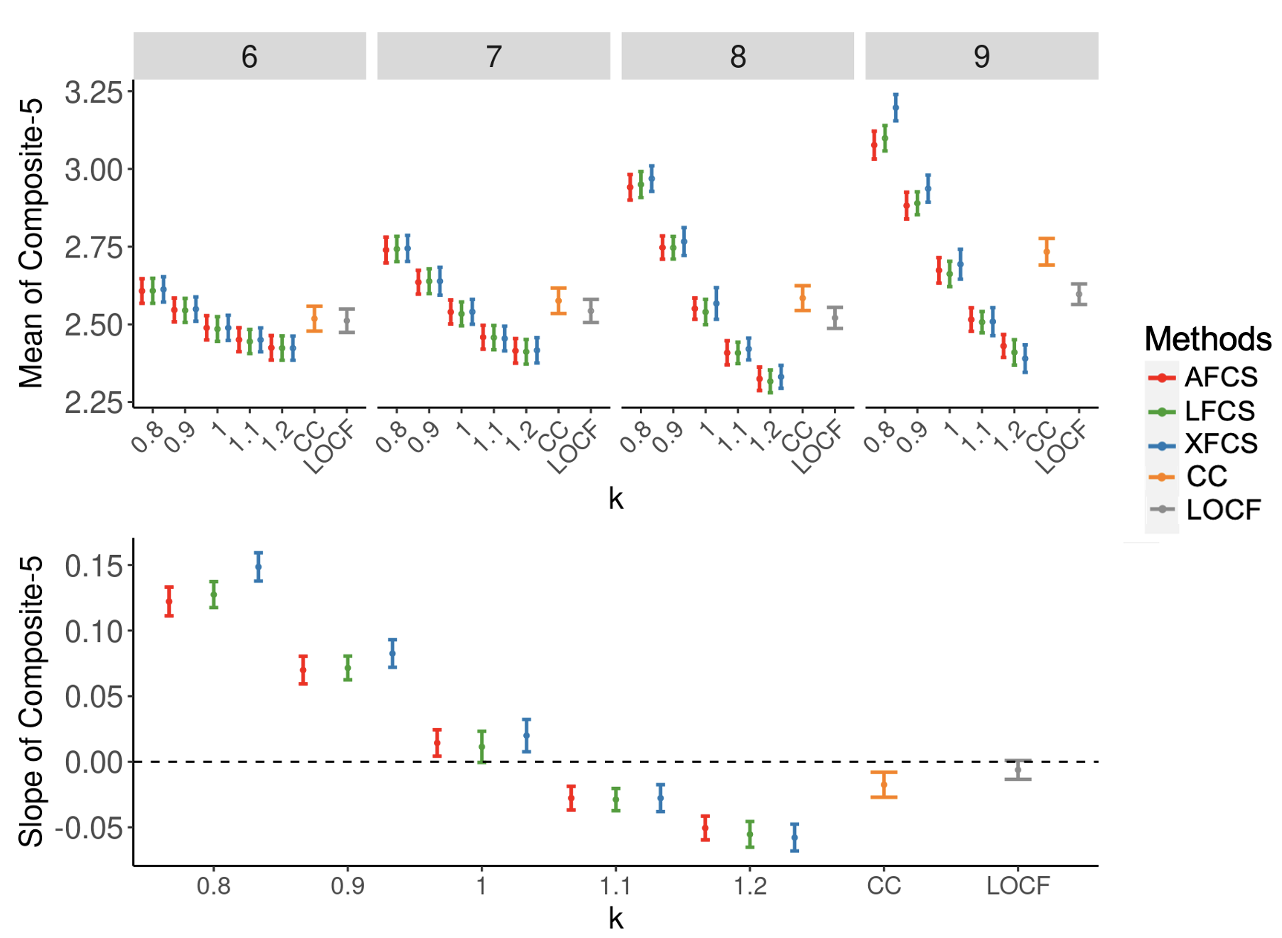
**

**Figure S9. Means and slopes of Composite-5 in the FHS Offspring cohort (sensitivity analyses).** In the top panel, the y-axis represents the mean values of Composite-5, and the x-axis represents different ignorability assumptions in two-stage MI. Each bar denotes the point estimate and its 95% CI for the mean value of Composite-5. Four subpanels illustrate four exams. In the bottom panel, the y-axis represents the slope values of Composite-5 across exams in the linear mixed effects models, and the x-axis represents different ignorability assumptions. Each bar denotes the point estimate and its 95% CI for the slope value of Composite-5. MI, multiple imputation; AFCS, all fully conditional specification; LFCS, longitudinal fully conditional specification; XFCS, cross-sectional fully conditional specification; CC, complete case analysis; LOCF, last observation carried forward; CI, confidence interval.

**
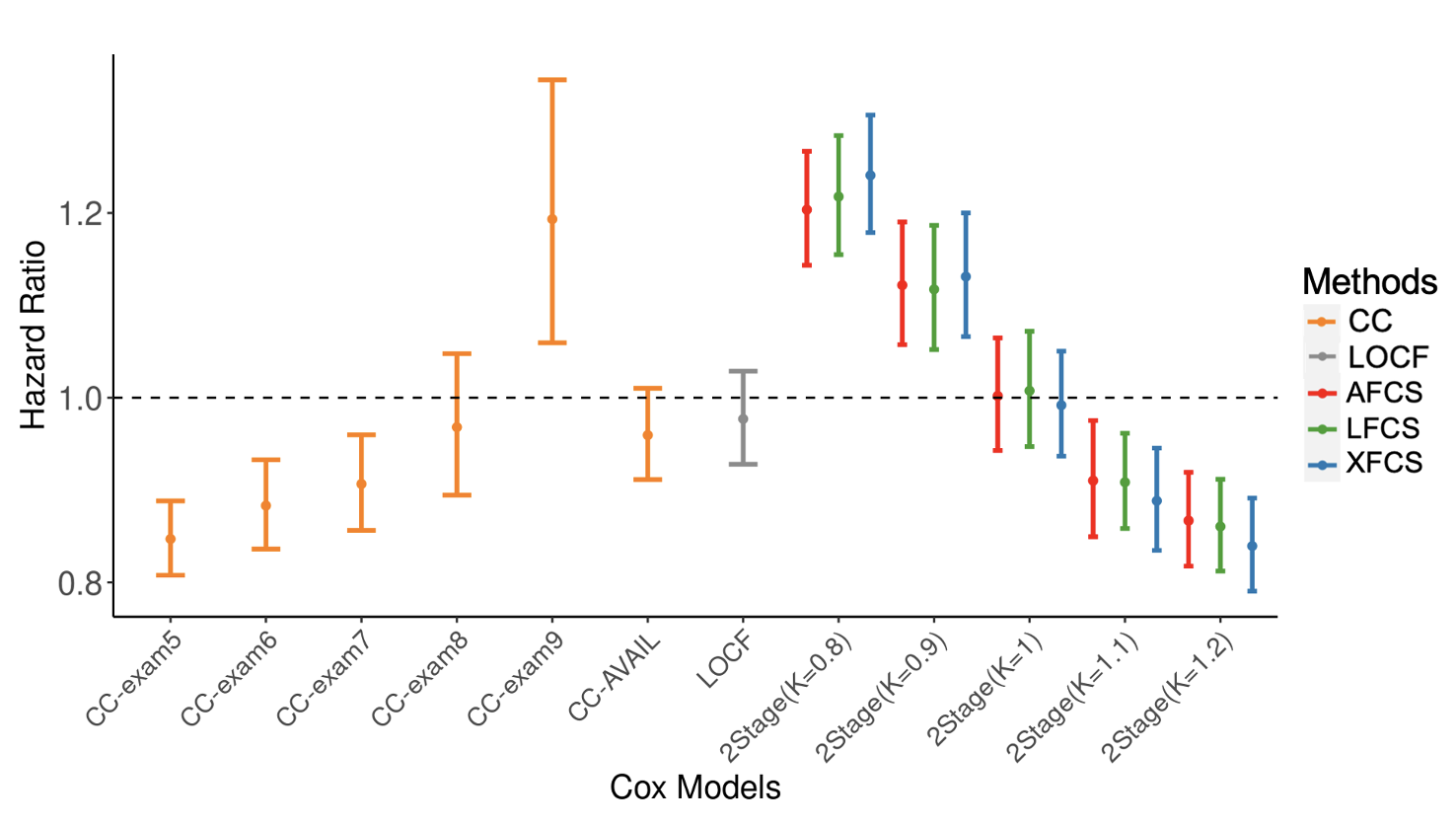
**

**Figure S10. HRs in the FHS Offspring cohort (sensitivity analyses).** The y-axis represents the HRs of Composite-5 in Cox models, and the x-axis represents different Cox models in Table 5. Each bar denotes the point estimate and its 95% CI for the HR of death for each unit increase in Composite-5. HR, hazard ratio; AFCS, all fully conditional specification; LFCS, longitudinal fully conditional specification; XFCS, cross-sectional fully conditional specification; CC, complete case analysis; LOCF, last observation carried forward; CI, confidence interval.
